# Scalable probabilistic PCA for large-scale genetic variation data

**DOI:** 10.1371/journal.pgen.1008773

**Published:** 2020-05-29

**Authors:** Aman Agrawal, Alec M. Chiu, Minh Le, Eran Halperin, Sriram Sankararaman

**Affiliations:** 1 Department of Computer Science, Indian Institute of Technology, Delhi, India; 2 Bioinformatics Interdepartmental Program, University of California, Los Angeles, California, United States of America; 3 Department of Computer Science, University of California, Los Angeles, California, United States of America; 4 Department of Human Genetics, University of California, Los Angeles, California, United States of America; 5 Department of Anesthesiology and Perioperative Medicine, University of California, Los Angeles, California, United States of America; 6 Department of Computational Medicine, David Geffen School of Medicine, University of California, Los Angeles, California, United States of America; 7 Institute of Precision Health, University of California, Los Angeles, California, United States of America; McGill University, CANADA

## Abstract

Principal component analysis (PCA) is a key tool for understanding population structure and controlling for population stratification in genome-wide association studies (GWAS). With the advent of large-scale datasets of genetic variation, there is a need for methods that can compute principal components (PCs) with scalable computational and memory requirements. We present ProPCA, a highly scalable method based on a probabilistic generative model, which computes the top PCs on genetic variation data efficiently. We applied ProPCA to compute the top five PCs on genotype data from the UK Biobank, consisting of 488,363 individuals and 146,671 SNPs, in about thirty minutes. To illustrate the utility of computing PCs in large samples, we leveraged the population structure inferred by ProPCA within White British individuals in the UK Biobank to identify several novel genome-wide signals of recent putative selection including missense mutations in *RPGRIP1L* and *TLR4*.

## Author summary

Principal component analysis is a commonly used technique for understanding population structure and genetic variation. With the advent of large-scale datasets that contain the genetic information of hundreds of thousands of individuals, there is a need for methods that can compute principal components (PCs) with scalable computational and memory requirements. In this study, we present ProPCA, a highly scalable statistical method to compute genetic PCs efficiently. We systematically evaluate the accuracy and scalability of our method on large-scale simulated data and apply it to the UK Biobank. Leveraging the population structure inferred by ProPCA within the White British individuals in the UK Biobank, we identify several novel signals of putative recent selection.

## Introduction

Inference of population structure is a key step in population genetic analyses [1] with applications that include understanding genetic ancestry [2–4] and controlling for confounding in genome-wide association studies (GWAS) [5]. While several methods have been proposed to infer population structure (e.g., [6–10]), principal component analysis (PCA) is one of the most widely used [6, 11]. Unfortunately, the naive approach for estimating principal components (PCs) by computing a full singular value decomposition (SVD) scales quadratically with sample size (for datasets where the number of SNPs is larger than sample size), resulting in runtimes unsuitable for large data sets.

In light of these challenges, several solutions have been proposed for the efficient computation of PCs. One approach taken by many recent scalable implementations (FastPCA [12], FlashPCA2 [13], bigsnpr [14], TeraPCA [15], PLINK2 [16]) takes advantage of the fact that typical applications of PCA in genetics only require computing a small number of top PCs; *e.g*. GWAS typically use 5-20 PCs to correct for stratification [17]. These methods can be grouped according to their underlying algorithm: blanczos (FastPCA, PLINK2, TeraPCA) or the implicitly restarted Arnoldi algorithm (FlashPCA2, bigsnpr). An alternative approach for efficient computation of PCs takes advantage of the parallel computation infrastructure of the cloud [18]. However, the cost of cloud usage is roughly proportional to the number of CPU hours used by these algorithms, making them cost-prohibitive. Finally, these scalable implementations lack a full probabilistic model, making them challenging to extend to settings with missing genotypes or linkage disequilibrium (LD) between SNPs.

In this work, we describe ProPCA, a scalable method to compute the top PCs on genotype data. ProPCA is based on a previously proposed probabilistic model [19, 20], of which PCA is a special case. While PCA treats the PCs and the PC scores as fixed parameters, probabilistic PCA imposes a prior on the PC scores. This formulation leads to an iterative Expectation Maximization (EM) algorithm for computing the PCs. ProPCA leverages the structure of genotype data to further reduce the computation time in each iteration of the EM algorithm. The EM algorithm requires only a small number of iterations to obtain accurate estimates of the PCs resulting in a highly scalable algorithm.

ProPCA obtains a computational speed-up through the integration of the Mailman algorithm [21] into its EM algorithm. The Mailman algorithm allows for fast matrix-vector multiplication when there are a finite number of values (e.g. genotypes) in exchange for additional memory usage. As a result, ProPCA requires more memory than some of the other scalable PCA methods. However, the increased memory consumption is reasonable; often still within the memory available within typical computing environments.

In both simulated and real data, ProPCA is able to accurately infer the top PCs while scaling favorably with increasing sample size. We applied ProPCA to compute the top five PCs on genotype data from the UK Biobank, consisting of 488,363 individuals and 146,671 SNPs, in less than thirty minutes. To illustrate how the ability to compute PCs in large samples can lead to biological discovery, we leveraged the population structure inferred by ProPCA within the White British individuals in the UK Biobank [22] to scan for SNPs that are not well-modeled by the top PCs and, consequently, identify several novel genome-wide signals of recent positive selection. Our scan recovers sixteen loci that are highly differentiated across the top five PCs that are likely signals of recent selection. While these loci include previously reported targets of selection [12], the larger sample size that we analyze here allows us to identify eleven novel signals including a missense mutation in *RPGRIP1L* (*p* = 2.09 × 10^−9^) and another in *TLR4* (*p* = 7.60 × 10^−12^).

A number of algorithms that analyze genotype data, including methods for heritability estimation and association testing, can be modeled as iterative procedures where the core computational operation is similar to that solved by ProPCA. Thus, the algorithm that we employ in this work can potentially lead to highly scalable algorithms for a broad set of population genetic analyses.

## Results

### Accuracy

We first assessed the accuracy of ProPCA using the simulation framework described in the Methods. We generated datasets containing 50, 000 SNPs and 10, 000 individuals across *q* populations, where *q* was chosen to be 5 and 10. The populations were simulated with varying levels of population differentiation that are typical of present-day human populations (values of *F*_*st*_ ranging from 0.001 to 0.01) and were small enough so that we could compute the full SVD thereby allowing us to estimate the accuracy of the PCs computed by ProPCA. To measure accuracy, we computed the mean of explained variances (MEV), a measure of the overlap between the subspaces spanned by the PCs estimated by ProPCA compared to the PCs computed using a full SVD (Methods). ProPCA, All methods are able to estimate highly accurate PCs (values of MEV close to 1) across the range of parameters (Table 1).

**Table 1:** ProPCA accurately estimates principal components relative to other methods.

*F*_*st*_	ProPCA		FlashPCA2		fastPCA		PLINK2		bigsnpr		TeraPCA	
	*K* = 5	*K* = 10	*K* = 5	*K* = 10	*K* = 5	*K* = 10	*K* = 5	*K* = 10	*K* = 5	*K* = 10	*K* = 5	*K* = 10
0.001	0.987	1.000	1.000	1.000	1.000	1.000	1.000	1.000	1.000	1.000	1.000	0.999
0.002	0.999	1.000	1.000	1.000	1.000	1.000	1.000	1.000	1.000	1.000	1.000	1.000
0.003	0.999	1.000	1.000	1.000	1.000	1.000	1.000	1.000	1.000	1.000	1.000	1.000
0.004	0.999	1.000	1.000	1.000	1.000	1.000	1.000	1.000	1.000	1.000	1.000	1.000
0.005	1.000	1.000	1.000	1.000	1.000	1.000	1.000	1.000	1.000	1.000	1.000	1.000
0.006	1.000	1.000	1.000	1.000	1.000	1.000	1.000	1.000	1.000	1.000	1.000	1.000
0.007	1.000	1.000	1.000	1.000	1.000	1.000	1.000	1.000	1.000	1.000	1.000	1.000
0.008	1.000	1.000	1.000	1.000	1.000	1.000	1.000	1.000	1.000	1.000	1.000	1.000
0.009	1.000	1.000	1.000	1.000	1.000	1.000	1.000	1.000	1.000	1.000	1.000	1.000
0.010	1.000	1.000	1.000	1.000	1.000	1.000	1.000	1.000	1.000	1.000	1.000	1.000

The principal components computed by ProPCA are compared to the PCs obtained from a full SVD on a genotype dataset containing 50, 000 SNPs and 10, 000 individuals. Accuracy was measured by the mean of explained variance (MEV) which measures the overlap between the set of PCs inferred from ProPCA and those from SVD across values of *F*_*st*_ ∈ {0.001, …, 0.01}. We report MEV for *K* = 5 using 5 populations as well as for *K* = 10 PCs using 10 populations. Methods shown are run using their default parameters.

### Runtime

We assessed the scalability of ProPCA with increasing sample size (Methods). We simulated genotypes from six populations containing 100, 000 SNPs and sample sizes varying from 10, 000 to 1, 000, 000 with *F*_*st*_ = 0.01.

**Fig 1:**
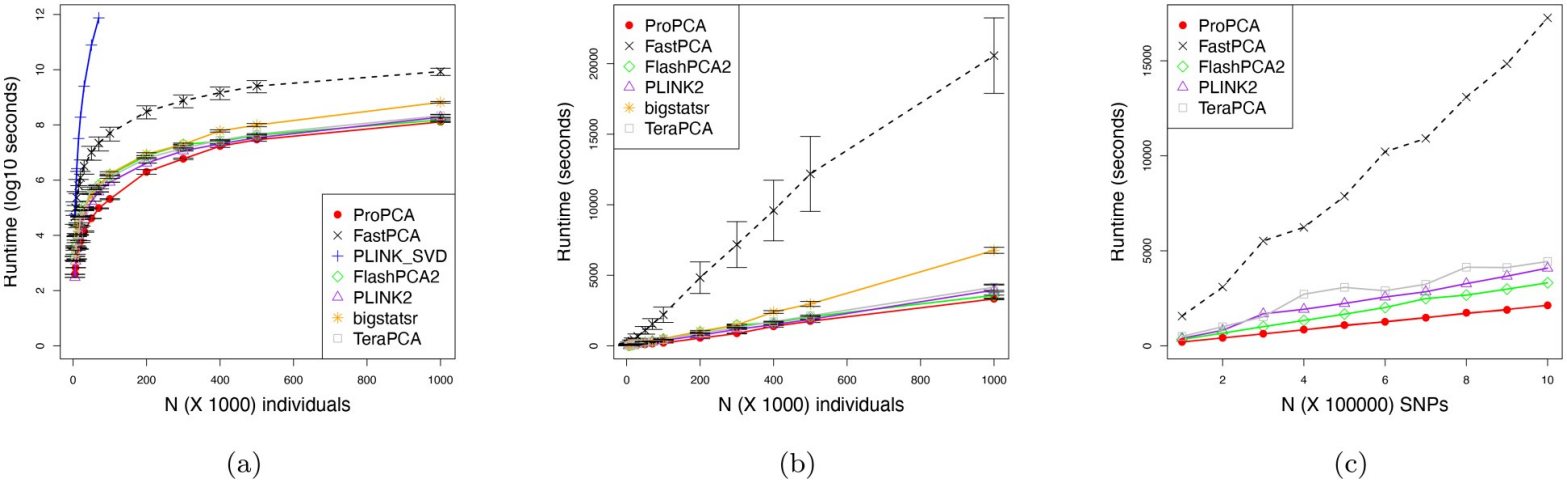
ProPCA is computationally efficient. Comparison of runtimes over simulated genotype data varied over individuals and SNPs. Figures 1a and 1b display the total runtime containing 100, 000 SNPs, six subpopulations, *F*_*st*_ = 0.01 and individuals varying from 10, 000 to 1, 000, 000. We report the mean and standard deviation over ten trials. Figure 1b compares the runtimes of all algorithms excluding PLINK_SVD which could only run successfully up to a sample size of 70, 000. Figure 1c displays the total runtime containing 100, 000 individuals, six subpopulations, *F*_*st*_ = 0.01, and SNPs varying from 10, 000 to 1, 000, 000. All methods were capped to a maximum of 100 hours and a maximum memory of 64 GB and run using default settings. We were unable to include bigstatsr in the SNP benchmark as it does not allow for monomorphic SNPs.

We compared the wall-clock time for running ProPCA, the SVD implementation in PLINK (PLINK_SVD [23]), FastPCA [12], FlashPCA2 [13], bigsnpr [14], PLINK2 [16], TeraPCA [15]). The SVD implementation in PLINK could not run in reasonable time on datasets exceeding 70, 000 individuals (Fig 1a). While all the other methods scale with sample size, ProPCA is faster than the methods compared against (Fig 1b). ProPCA computes PCs in about 30 minutes even on the largest data containing a million individuals and 100, 000 SNPs. We similarly explored how each method scale in terms of the number of variants. We repeated our experiment by varying the number of SNPs from 10, 000 to 1, 000, 000 while keeping the sample size constant at 100, 000 and found similar results (Fig 1c).

We further tested runtime as a function of the number of PCs on a simulated dataset containing 10,000 individuals, 50,000 SNPs, and 20 latent populations separated at *F*_*ST*_ = 0.01. We find that PLINK2 and ProPCA scale linearly when computing the upto the top 40 PCs (S1 Fig). FlashPCA2 is efficient at computing 2-20 PCs, but increases in runtime when computing a single PC or more than 20 PCs. We found that this trend was both reproducible across different datasets. We also tested bigsnpr/bigstatsr, which uses the same underlying algorithm as FlashPCA2 and found a similar trend, *i.e*., its performance to be similar to FlashPCA2 in that it is efficient at computing 1-20 PCs, but we see a steady increase in runtime after 20 PCs.

We tested a final scenario in which each method computed 40 PCs on our two largest simulated datasets containing one million SNPs and 10,000 individuals dataset as well as the one million individuals and 10,000 SNPs dataset (Table 2). We find that ProPCA can compute the 40 PCs most efficiently under two days for both datasets while other methods required 2-4 days.

**Table 2:** Runtimes of methods on largest simulated datasets for 40 principal components.

Method	SNPs	Individuals
**bigstatsr**	-	103
**FastPCA**	-	-
**FlashPCA2**	93	114
**PLINK2**	74	72
**ProPCA**	35	28
**TeraPCA**	49	48

We computed 40 PCs from each method on each of our largest simulated datasets. Times are reported in hours. The ‘SNPs’ column contains the runtime on a 1 million SNP and 10,000 individuals dataset while the ‘Individuals’ contains the runtime on a 1 million individual and 10,000 SNP dataset. FastPCA could not be run to completion on either dataset due to a segmentation fault while bigstatsr could not run on the SNPs dataset due to the inclusion of monomorphic SNPs. All methods were run with default parameters except TeraPCA, which was run with ‘-rfetched 4000’ for the SNPs dataset and ‘-rfetched 2000’ for the Individuals dataset due to a segementation fault.

Since ProPCA, FastPCA, and FlashPCA2 are all based on iterative algorithms, their runtimes depend on details of convergence criterion. We performed an additional experiment to compare the runtime of ProPCA, FastPCA (for which we could instrument the source code) for a single iteration and found ProPCA to be three to four times faster than FastPCA across the range of sample sizes (S2 Fig).

Measuring the accuracy of the PCs (MEV) as a function of runtime (on datasets with a range of *F*_*st*_ containing 50, 000 SNPs and 10, 000 individuals so that we could compare the estimated PCs to exact PCs), ProPCA attains a given MEV in about half the time as FastPCA and FlashPCA2 (S3 Fig).

### Memory

We assessed the memory usage of ProPCA and other methods as a function of individuals and SNPs (S4a and S4b Fig). Due to computations utilized by the Mailman algorithm, ProPCA uses more memory than other methods, but is still relatively efficient requiring about 40 GB on the largest dataset. Memory usage for ProPCA scales linearly with respect both individuals and SNPs.

### Application to real genotype data

**Fig 2:**
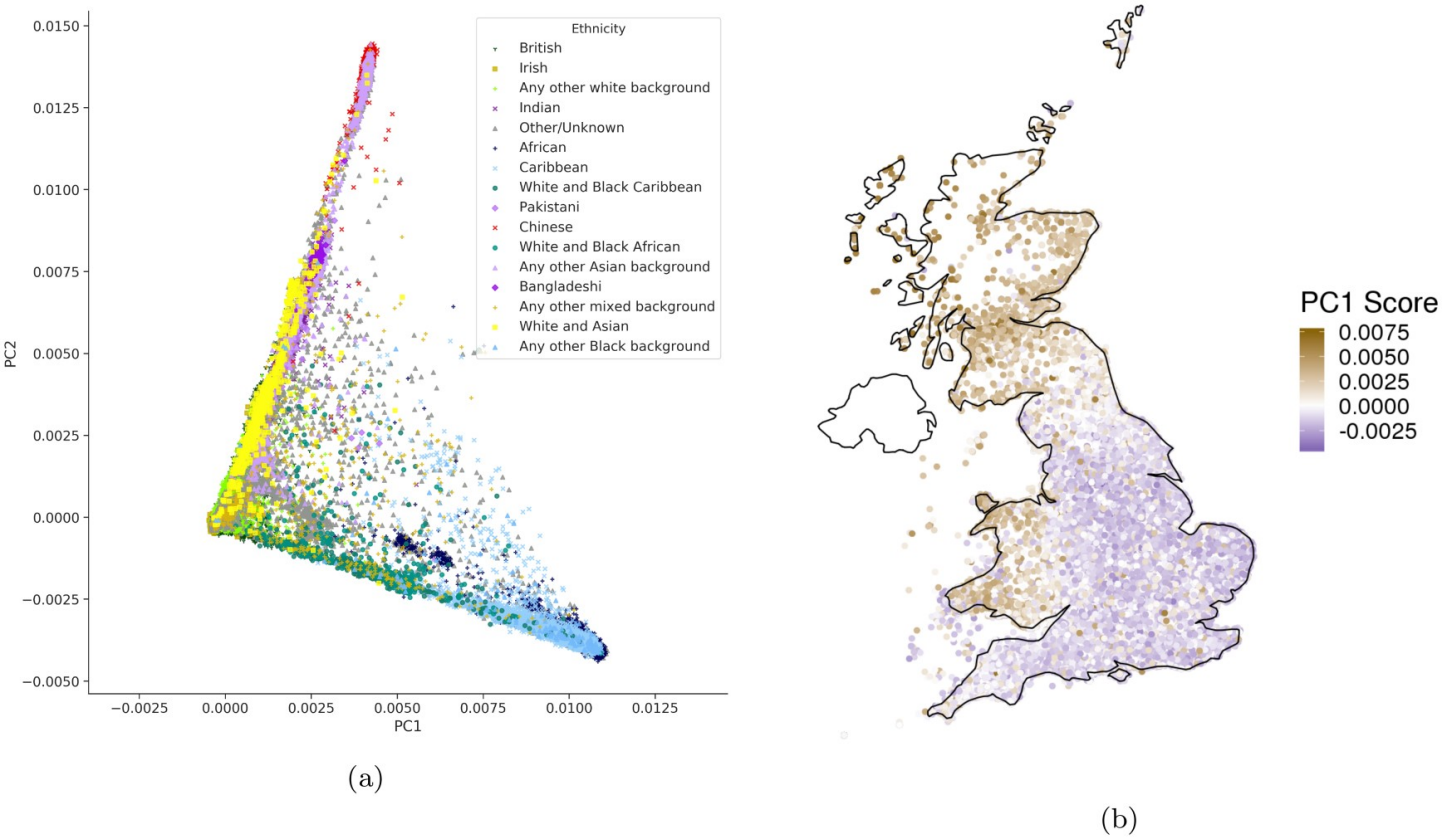
Principal components uncover population and geographic structure in the UK Biobank. We used ProPCA to compute PCs on the UK Biobank data. Figure 2a shows the first two principal components to reveal population structure. Figure 2b shows geographic structure by plotting the score of 276, 736 unrelated White British individuals on the first principal component on their birth location coordinates.

We applied ProPCA to genotype data from Phase 1 of the 1000 Genomes project [24]. On a dataset of 1092 individuals and 442, 350 SNPs, ProPCA computes the top forty PCs that are qualitatively indistinguishable from running a full SVD (S5 Fig). Furthermore, we tested each method’s ability to compute 5-40 PCs on this dataset. We took a small subset for 450k SNPs and 1,092 individuals for which we could compute the full SVD. We tested all methods at increments of 5 PCs to 40 PCs and ultimately found that all that all methods still performed well across the range tested (MEV ≥ 0.95) (S1 Table). We also applied ProPCA to genotype data from the UK Biobank [22] consisting of 488, 363 individuals and 146, 671 SNPs after QC. ProPCA can compute the top five PCs in about 30 minutes and the resulting PCs reflect population structure within the UK Biobank, consistent with previous studies [22] (Fig 2a).

### Application to scans for selection

Since the PCs in ProPCA are computed as maximum likelihood estimates under a probabilistic model, ProPCA provides a natural framework for applications such as hypothesis testing. By utilizing the statistical assumptions and set up provided by the ProPCA model, we developed a statistical test to search for SNPs that are not well-modeled by the ProPCA model as a means of discovering signals of natural selection (Methods and S1 Text). This statistic relies on the observation that a SNP evolving under positive selection is expected to exhibit differentiation in the frequencies of its alleles that is extreme compared to a typical SNP that is evolving neutrally [25].

Since deviations from the ProPCA model can occur due to reasons unrelated to selection, we filtered out SNPs with high rates of missingness, low minor allele frequency (MAF), and presence in regions of long-range LD [26] (Methods). We ran ProPCA to infer the top five PCs on 276, 736 unrelated White British samples and the UK Biobank SNP set consisting of 146, 671 SNPs obtained by further removing SNPs in high LD (S6 Fig).

The Pearson correlation coefficient between birth location coordinates and the PC score for each individual reveals that the estimated PCs capture geographic structure within the UK (Fig 2b, S7 Fig, S2 Table). We used these PCs to perform a selection scan on a larger set of 516, 140 SNPs and we report SNPs that are genome-wide significant after accounting for the number of SNPs as well as PCs tested (p-value $<\frac{0.05}{6\times 516,140}$; we use 6 to account for the additional combined test statistic that we describe later). We ensured that the selection statistic for each PC was well-calibrated against a $\chi_{1}^{2}$ distribution (S8 Fig) and genomic inflation (λ_*GC*_) values for each of the PCs showed no substantial inflation (S3 Table). While our statistic is closely related to a previously proposed statistic to detect selection on PCs (S1 Text), we found that our proposed statistic is better calibrated (S3 Table).

**Fig 3:**
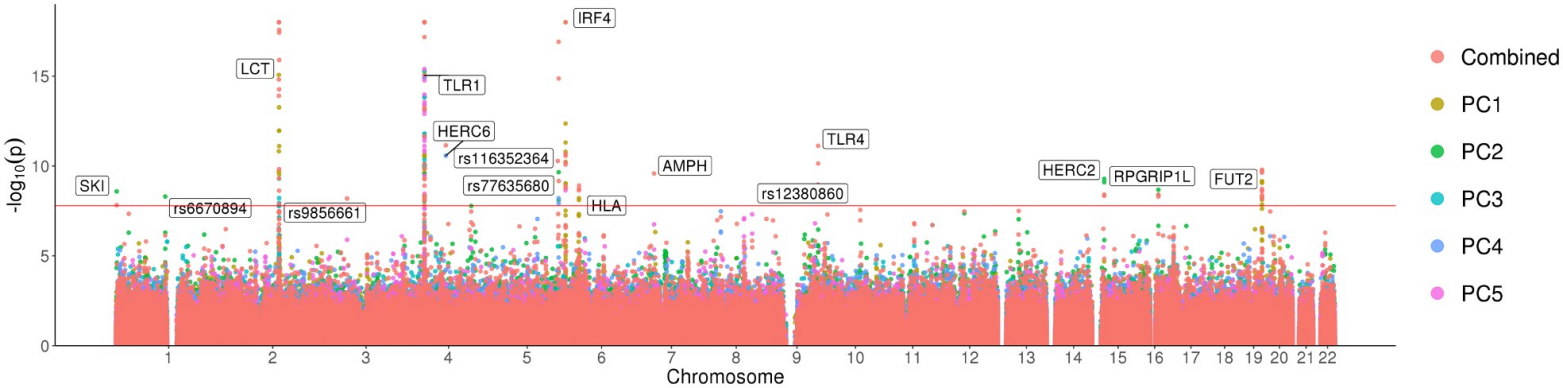
Selection scan for the first five principal components in the white British individuals in the UK Biobank. A Manhattan plot with the −log_10_ *p* values associated with the test of selection displayed for the first five principal components for the unrelated White British subset of the UK Biobank. The red line represents the Bonferroni adjusted significance level (*α* = 0.05). Significant loci are labeled. Signals above −log_10_(*p*) = 18 were capped at this value for better visualization.

Our scan revealed a total of 59 SNPs that were genome-wide significant (S4 Table). Clustering these signals into 1 Mb windows centered around the most significant SNP for each PC, we obtained twelve non-overlapping loci that contain putative targets of selection (Fig 3, S5 Table). These twelve loci include five that were previously reported to be signals of selection in the UK with genome-wide significance: *LCT* (*rs7570971* with *p* = 8.51 × 10^−16^), *TLR1* (*rs5743614*, *p* = 5.65 × 10^−25^), *IRF4* (*rs62389423*, *p* = 8.80 × 10^−42^), *HLA* (*rs9267817*, *p* = ×6.17 × 10^−9^), and *FUT2* (*rs492602*, *p* = 7.02 × 10^−10^) [12]. The larger sample size that we analyze here also reveals novel signals at additional loci. Four of the twelve signals were previously suggested to be signals of selection but were not genome-wide significant: *HERC2* (*rs12913832*, *p* = 5.21 × 10^−10^), *RPGRIP1L* (*rs61747071*, *p* = 2.09 × 10^−9^), *SKI* (*rs79907870*, *p* = 2.58 × 10^−9^), *rs77635680* (*p* = 2.22 × 10^−10^) [12] while the remaining three loci: *HERC6* (*rs112873858*, *p* = 2.68 × 10^−11^), *rs6670894* (*p* = 4.98 × 10^−9^), and *rs12380860* (*p* = 8.62 × 10^−9^) appear to be previously unreported.

To validate our findings, we utilized birth location coordinates for each individual and assigned them to geographical regions in the UK as defined in the Nomenclature of Territorial Units for Statistics level 3 (NUTS3) classification. We performed a test of association between the allele frequency of the top SNP in each of our novel loci with geographical regions and confirmed that SNPs identified in our selection scan show differences in allele frequencies across specific geographical regions (S6 Table).

One of the novel genome-wide significant loci is *RPGRIP1L*. *RPGRIP1L* is a highly conserved gene that encodes a protein that localizes at primary cilia and is important in development [27]. Mutations in this gene have been implicated with neurological disorders such as Joubert syndrome and Meckel syndrome [28], conditions that sometimes also result in additional symptoms such as eye diseases and kidney disorders [29]. The SNP with the most significant p-value in our scan in *RPGRIP1L*, *rs61747071*, is a missense loss-of-function mutation A229T that has been shown to lead to photoreceptor loss in ciliopathies [30].

We created an additional variant of our selection statistic which tests for SNPs that are not well-modeled by a linear combination of the first five PCs by summing the per-PC $\chi_{1}^{2}$ statistics resulting in a new chi-squared statistic with five degrees of freedom. Combining signals across PCs has been previously shown to boost power in association testing [31]. We verified that the resulting combined statistic is also calibrated (S8 Fig, S3 Table). Under this combined statistic, we recover majority of the loci found on each individual PC, but we also discover four additional novel loci: *AMPH* (*rs118079376*, *p* = 2.64 × 10^−10^), *TLR4* (*rs4986790*, *p* = 7.60 × 10^−12^), *rs9856661* (*p* = 6.46 × 10^−9^), and *rs116352364* (*p* = 5.24 × 10^−11^) (S7 Table).

*TLR4* is a member of the toll-like receptor family. The *TLR* gene family is known to play a fundamental role in pathogen recognition and activation of innate immunity, but *TLR4* in particular is involved with proinflammatory cytokines and has a pro-carcinogenic function [32]. The SNP with the most significant *p*-value at our *TLR*4 locus is *rs4986790*, a missense D299G mutation and D259G mutation on two different transcripts for the *TLR4* gene. The D299G mutation is of particular interest as this mutation is strongly correlated with increased infection by *Plasmodium falciparum*, a parasite that causes malaria [33, 34].

To better understand the signals of selection that the proposed statistic is sensitive to, we compared the time-scale for our selection hits to those from a recent study that is designed to detect recent positive selection [35] (S9 Fig). Using estimates of allelic ages for variants in the 1000 Genomes Project [36], we find that the variants detected by the proposed statistic tend to be older on average than those found to have a singleton-density score > 4 from Field *et al*. 2016 (average age of 19, 007 generations for our statistic vs 11, 944 generations for the SDS statistic using the combined mutation and recombination clock). We caution that the interpretation of these results is complicate by the considerable uncertainty in the allelic age estimates. Further, the timing of an episode of selection might post-date the age of the mutation—for example, when selection acts on standing variation. Finally, there is substantial variation in the mean age estimates of the hits. While the average age is around 19, 000 generations, 17 of the 42 putatively selected variants have ages less than 5, 000 generations. This suggests that the proposed statistic could be sensitive to both recent and older selection where the resulting allele frequencies are not well-modeled by the PCs.

To further illustrate how the ability to compute PCs in large samples is necessary for biological discovery, we analyzed how many selection signals we discover as a function of sample size by randomly subsampling the number of individuals from the White British population and repeating our analyses (S10 Fig). We ultimately find that sample sizes larger than 150, 000 individuals are required to retain over 80% of the total signals of selection we discover.

## Discussion

We have presented, ProPCA, a scalable method for PCA on genotype data that relies on performing inference in a probabilistic model. Inference in this model consists of an iterative procedure that uses a fast matrix-vector multiplication algorithm. We have demonstrated its accuracy and efficiency across diverse settings. Further, we have demonstrated that ProPCA can accurately estimate population structure within the UK Biobank dataset and how this structure can be leveraged to identify targets of recent putative selection.

The algorithm that we employ here to accelerate the EM updates is of independent interest. Beyond PCA, several algorithms that operate on genotype data perform repeated matrix-vector multiplication on the matrix of genotypes. For example, association tests and permutation tests, can be formulated as computing a matrix-vector product where the matrix is the genotype matrix while the vector consists of phenotype measurements. Indeed, the algorithm has been used to accelerate heritability estimation [37]. The idea that SVD computations can leverage fast matrix-vector multiplication operations to obtain computational efficiency is well known in the numerical linear algebra literature [38]. Indeed, the algorithms [13, 38] implemented in other PCA methods can also utilize these ideas to gain additional computational efficiency. Alternate approaches to improve matrix-vector multiplication in the genetics setting include approaches that rely on sparsity of the genotype matrix. It is important to note that the speedup obtained from the Mailman algorithm does not rely explicitly on sparsity and could be applied even to dense matrices. It would be of interest to contrast the use of sparse multiplication versus the Mailman algorithm and to investigate the potential to combine these two approaches to be able to leverage sparsity as well as the discrete nature of the genotype matrix.

It is likely that different algorithms and implementations to compute PCs (and more generally, infer population structure) might be appropriate based on the specific application. The choice of the specific algorithm and implementation involves a number of trade-offs. While ProPCA is computationally efficient, its use of the Mailman algorithm results in a bigger memory footprint relative to other methods. The probabilistic formulation underlying ProPCA allows the algorithm to be generalized in several directions. One direction is the application of PCA in the presence of missing data that often arises when analyzing multiple datasets. We have explored an extension of the ProPCA model to this setting (S1 Text, S11 Fig). While this approach is promising, a limitation of the use of the Mailman algorithm within ProPCA is the requirement of discrete genotypes, which prevents ProPCA from being directly applied to dosages (e.g. imputed genotypes). Another potential future direction is in modeling linkage disequilibrium and in incorporating rare variants which have the potential to reveal structure that is not apparent from the analysis of common SNPs [39, 40]. Current applications of PCA remove correlated SNPs and singletons though this has been shown to discard information [12]. One possible way to incorporate LD would leverage the connection between haplotype copying models [41] and the multivariate normal model of PCA [42], or by a whitening transformation [4]. Further, the observation model can also be modified to account for the discrete nature of genotypes [3, 43]. A number of non-linear dimensionality reduction methods have been recently proposed [44, 45]. A comparison of these methods to ProPCA (in terms of statistical structure that the methods aim to detect, ability to handle missing data, and computational scalability) would be of great interest. Finally, leveraging fine-scale population structure inferred from large-scale data to study recent positive selection in human history is an important direction for future work. While the probabilistic model underlying ProPCA leads to a natural model for testing for selection, other hypotheses about models of selection could lead to other tests of selection. The challenge is to design realistic statistical models of population structure while enabling inference at scale.

### Software availability

ProPCA is available at https://github.com/sriramlab/ProPCA.

## Materials and methods

### Principal components analysis (PCA)

We observe genotypes from *n* individuals at *m* SNPs. The genotype vector for individual *i* is a length *m* vector denoted by ***g***_*i*_ ∈ {0, 1, 2}^*m*^. The *j*^*th*^ entry of ***g***_*i*_ denotes the number of minor allele carried by individual *i* at SNP *j*. Let ***G*** be the *m* × *n* genotype matrix where ***G*** = [***g***_1_…***g***_*n*_]. Let ***Y*** denote the matrix of standardized genotypes obtained by centering and rescaling each row of the genotype matrix ***G*** so that ∑_*j*_ *y*_*i*,*j*_ = 0 and $\sum_{j}y_{i,j}^{2}=1$ for all *i* ∈ {1, …, *m*}.

Principal components analysis (PCA) [11] attempts to find a low-dimensional linear transformation of the data that maximizes the projected variance or, equivalently, minimizes the reconstruction error. Given the *m* × *n* matrix ***Y*** of standardized genotypes and a target dimension *k*, PCA attempts to find a *m* × *k* matrix with orthonormal columns ***W*** and *n* × *k* matrix ***Z*** that minimizes the reconstruction error: ‖***Y*** − ***WZ***^T^‖_*F*_ where ${\left\|A\right\|}_{F}=\sqrt{\sum_{i,j}A_{i,j}^{2}}$ is the Frobenius norm of the matrix ***A***. To solve the PCA problem, we perform a singular-value decomposition (SVD) of the standardized genotype matrix ***Y*** = ***U***Σ***V***^T^ and set $\hat{W}=U_{K}$, where ***U***_*K*_ is a *m* × *k* matrix containing the *k* columns of ***U*** corresponding to the *k* largest singular vectors of ***Y***.

### Probabilistic PCA

PCA can be viewed as a limiting case of the probabilistic PCA model [10, 19, 20]. Probabilistic PCA models the observed data $y_{i}\in R^{m},i\in \left\{1\;\ldots ,n\right\}$ as a linear transformation of a *k*-dimensional latent random variable ***x***_*i*_ (*k* ≤ *m*) with additive Gaussian noise. Denoting the linear transformation by the *m* × *k* matrix ***C***, and the (*m*-dimensional) noise by ***ϵ***_*i*_ (with isotropic covariance matrix *σ*^2^***I***_*m*_), the generative model can be written as

$$\begin{array}{ccc} y_{i}|x_{i};\epsilon_{i} & = & Cx_{i}+\epsilon_{i}\\ x_{i} & \overset{iid}{∼} & N(0,I_{k})\\ \epsilon_{i} & \overset{iid}{∼} & N(0,\sigma^{2}I_{m}) \end{array}$$ (1)

The maximum likelihood estimate of the matrix ***C*** in this model has been shown to span the *k*-dimensional principal subspace of the data ***Y*** = [***y***_1_,…***y***_*n*_] [46].

### EM algorithm for PCA

Since probabilistic PCA is a probabilistic model endowed with latent variables, the EM algorithm presents a natural approach to compute the maximum likelihood estimates of the model parameters (***C***, *σ*^2^) [19, 20]. The EM algorithm for learning the principal components can be derived as a special case of the EM algorithm for the probabilistic PCA model where the variance of the observation noise *σ*^2^ tends to zero leading to these updates:

$$\begin{array}{c} \text{E-step}:\;X={\left(C^{T}C\right)}^{-1}C^{T}Y \end{array}$$ (2)

$$\begin{array}{c} \text{M-step}:\;C=YX^{T}{\left(XX^{T}\right)}^{-1} \end{array}$$ (3)

Here ***X*** = [***x***_1_…***x***_*n*_] is a *k* × *n* matrix and ***Y*** = [***y***_1_…***y***_*n*_] is a *m* × *n* matrix. Noting that all matrix inversions require inverting a *k* × *k* matrix, the computational complexity of the E-step is $O(k^{2}m+k^{3}+k^{2}m+mnk)$ while the computational complexity of the M-step is $O(k^{2}n+k^{3}+k^{2}n+mnk)$. For small *k* and large *m*, *n*, the per-iteration runtime complexity is O(mnk). Thus, the EM algorithm provides a computationally efficient estimator of the top *k* PCs when the number of PCs to be estimated is small.

### Sub-linear time EM

The key bottleneck in the EM algorithm is the multiplication of the matrix ***Y*** with matrices ***E*** = (***C***^*T*^***C***)^−1^***C***^*T*^ and ***M*** = ***X***^*T*^(***XX***^*T*^)^−1^.

The vectors representing the sample mean and standard deviation of the genotypes at each SNP are denoted $\bar{g}$ and *s*. Assuming no entry in *s* is zero (we remove SNPs that have no variation across samples), the matrix of standardized genotypes ***Y*** can be written as:

$$Y=diag{\left(s\right)}^{-1}G-\rho 1_{n}^{\text{T}}$$

Here *diag*(***x***) is an operator that constructs a diagonal matrix with the entries of ***x*** along its diagonals, **1**_*n*_ is a length *n* vector with each entry equal to one, and ***ρ*** is a length *m* vector with $\rho_{j}=\frac{{\bar{g}}_{j}}{s_{j}}$, *j* ∈ {1, …, *m*}.

The EM updates can be written as:

$$\begin{array}{ccc} X & = & EY=E\;\text{diag}{\left(s\right)}^{-1}G-E\rho 1_{n}^{\text{T}}\\ & = & \tilde{E}G-E\rho 1_{n}^{\text{T}} \end{array}$$ (4)

$$\begin{array}{ccc} C & = & YM={diag(s)}^{-1}GM-\rho 1_{n}^{\text{T}}M \end{array}$$ (5)

Here $\tilde{E}$ can be computed in time O(km) while $E\rho 1_{n}^{\text{T}}$ and $\rho 1_{n}^{\text{T}}M$ can be computed in time O(nk+mk).

The key bottleneck in the E-step is the multiplication of the genotype matrix ***G*** by each of the *k* rows of the matrix $\tilde{E}$ and in the M-step, multiplication of ***G*** by each of the *k* columns of the matrix ***M*** respectively. Leveraging the fact that each element of the genotype matrix ***G*** takes values in the set {0, 1, 2}, we can improve the complexity of these multiplication operations from O(nmk) to $O(\frac{nmk}{\max({\text{log}}_{3}\;n,{\text{log}}_{3}\;m)})$ by extending the Mailman Algorithm [21]. For additional implementation details, see S1 Text.

### The Mailman algorithm

In the M-step, we need to compute ***c*** = ***Ab*** for an arbitrary real-valued vector ***b*** and a *m* × *n* matrix ***A*** whose entries take values in {0, 1, 2}. We assume that *m* = ⌈log_3_(*n*)⌉. Naive matrix-vector multiplication takes $O(\left\lceil {\text{log}}_{3}\left(n\right)\right\rceil n)$ time.

The Mailman algorithm decomposes ***A*** as ***A*** = ***U***_*n*_***P***. Here ***U***_*n*_ is the *m* × *r* matrix whose columns containing all *r* = 3^*m*^ possible vectors over {0, 1, 2} of length *m*. We set an entry *P*_*i*,*j*_ to 1 if column *j* of ***A*** matches column *i* of $U_{n}:\;A^{\left(j\right)}=U_{n}^{\left(i\right)}$. The decomposition of any matrix ***A*** into ***U***_*n*_ and ***P*** can be done in O(nm) time. Given this decomposition, the desired product ***c*** is computed in two steps, each of which has O(n) time complexity [21]:

$$\begin{array}{c} d=Pb,\;c=U_{n}d \end{array}$$

The Mailman algorithm provides computational savings in a setting where the cost of computing the decomposition of ***A*** are offset by the gains in repeated multiplication involving ***A***.

Similarly, in the E-step, we need to compute ***f***^T^***A*** in $O(\left\lceil {\text{log}}_{3}\left(n\right)\right\rceil n)$ time by computing ***A***^T^***f*** and computing a decomposition of ***A***^T^. A drawback of this approach is the need to store both decompositions that would double the memory requirements of the algorithm. Instead, we propose a novel variant of the Mailman algorithm that can compute ***f***^T^***A*** in $O(\left\lceil {\text{log}}_{3}\left(n\right)\right\rceil n)$ time using the same decomposition as **A** (S1 Text).

Additional details on efficient implementation of the EM and Mailman algorithms can be found in S1 Text.

### Simulations

We simulated genotypes at *m* independent SNPs across *n* individuals in which a single ancestral population diverged into *q* sub-populations with drift proportional to the *F*_*st*_, a measure of population differentiation. The allele frequency at SNP *f*_*j*,0_, *j* ∈ {1, …, *m*} in the ancestral population was sampled from a uniform distribution such that $f_{j,0}\overset{iid}{∼}Unif\left(0.05,0.95\right)$. Allele frequencies in each of the *l* subpopulations were generated by simulating neutral drift from the ancestral allele frequency, $f_{j,l}∼N\left(f_{j,0},f_{j,0}\left(1-f_{j,0}\right)F_{st}\right),l\in \left\{1,\ldots ,q\right\}$ and were set to 0 or 1 if they fell outside the interval [0, 1]. The genotypes of an individual in population *l* at SNP *j* was sampled from a *Binomial*(2, *f*_*j*,*l*_) distribution.

### Benchmarking

To compare estimated PCs to reference PCs, we computed the mean of explained variance (MEV)—a measure of the overlap between the subspaces spanned by the two sets of PCs. Two different sets of *K* principal components each produce a K-dimensional column space. A metric for the performance of a PCA algorithm against some baseline is to see how much the column spaces overlap. This is done by projecting the eigenvectors of one subspace onto the other and finding the mean lengths of the projected eigenvectors. If we have a reference set of PCs (*v*_1_, *v*_2_, …, *v*_*k*_) against which we wish to evaluate the performance of a set of estimated PCs (*u*_1_, *u*_2_, …, *u*_*k*_), $MEV=\frac{1}{k}\sum_{i=1}^{k}\sqrt{\sum_{j=1}^{k}{\left(v_{i}\cdot u_{j}\right)}^{2}}=\frac{1}{k}\sum_{i=1}^{k}\left\|U^{T}v_{i}\right\|$ where **U** is a matrix whose column vectors are the PCs which we are testing.

In practice, when attempting to compute the top *k* PCs, ProPCA was found to converge faster by computing *l* PCs for *l* > *k* PCs and retaining the top *k* PCs. The reason for this is that in the initial iterations of the EM algorithm, the estimates of the top PCs are noisy. We set *l* = *k* in our experiments for an effective 2*k*. While ProPCA could be run to convergence, we found that running it for *k* iterations already gave accurate results across the range of parameters considered. Our empirical results are consistent with our theoretical result that the EM algorithm converges exponentially fast in the spectral norm of the error matrix [38, 47] (S1 Text).

We compared ProPCA to the current state-of-the-art methods for computing PCs from genotype data: the SVD implementation in PLINK (PLINK_SVD [23]), FastPCA [12], FlashPCA2 [13], bigsnpr [14], PLINK2 [16], and TeraPCA [15]. PLINK_SVD refers to an exact computation of PCs using the full Singular Value Decomposition as implemented in the PLINK package (PLINK_SVD). FastPCA [12] is an implementation of the blanczos method, a randomized subspace iteration method [38] while FlashPCA2 [13] is an implementation of the implicitly restarted Arnoldi method [48]. PLINK2 [16] and TeraPCA [15] are reimplimentations of the FastPCA algorithm while bigsnpr [14] is a reimplimentation of the FlashPCA2 algorithm designed to utilize disk space as a file backend. We used default parameters for all methods unless otherwise stated. For benchmarking of bigsnpr, we included the creation of the file backend in timing as it is required to run any of the computations included in the backend. Furthermore, we excluded bigsnpr from some experiments due to the inability of its PCA function to natively handle missing data and when faced with monomorphic SNPs. All experiments were performed on a Intel(R) Xeon(R) CPU 2.10GHz server with 128 GB RAM, restricted to a single core, capped to a maximum runtime of 100 hours and a maximum memory of 64 GB.

### Selection scan

The White British cohort was identified by the UK Biobank as participants who self-identified as ‘British’ within the broader-level group ‘White’ while having similar ancestral background [22]. For our selection scan, we further filtered the 409, 634 individuals in the White British subset to obtain an unrelated White British subset by removing individuals with one other related individual in the data set (individuals with kinship coefficients greater than 0.0625 (third-degree relatedness) to any other individual as determined by KING [49]). After removing these individuals, we obtained an unrelated White British subset containing 276, 736 individuals.

We inferred the top five PCs using ProPCA on all 276, 736 unrelated White British individuals and a filtered SNP set containing 146, 671 SNPs (UK Biobank SNP set). SNPs in the UK Biobank SNP set consist of SNPs on the UK Biobank Axiom array from which SNPs were removed if they have missing rates greater than 1.5%, minor allele frequencies (MAF) less than 1%, or if they were in regions of long-range linkage disequilibrium. The remaining SNPs were then pruned for pairwise *r*^2^ less than 0.1 using windows of 1000 base pairs (bp) and a step-size of 80 bp.

We developed a selection statistic to search for SNPs whose variation is not well-explained by the ProPCA model, closely related to the selection statistic proposed in [12] (S1 Text). Under the probabilistic PCA model, the normalized genotype matrix is modeled by a low rank approximation and Gaussian noise, **Y** = **CX** + ***ϵ***. Given our low rank approximation of the genotype matrix, $\hat{Y}=\text{CX}$, we have the residual: $Y-\hat{Y}=\epsilon$. For a SNP *j*, the Gaussian noise, $\epsilon_{j}∼N\left(0,\sigma^{2}I_{n}\right)$. Projecting this residual onto a PC results in a univariate Gaussian with zero mean and constant variance across SNPs. This variance can be estimated as the sample variance ${\hat{\sigma }}^{2}$ of the resulting statistics across SNPs. In summary, we propose the statistic: $\frac{{\left({\left(y_{j}-{\hat{y}}_{j}\right)}^{T}x_{k}\right)}^{2}}{{\hat{\sigma }}^{2}}∼\chi_{1}^{2}$ for SNP *j*, given the *k*-th PC. The projection of the residual onto a PC allows the signal of selection to be interpreted in the context of deviations from ancestry captured by the specific PC.

Furthermore, a variant of this statistic, which we call the combined statistic, can be generated from the selection statistics computed on each individual PC using the observation that the resulting chi-squared statistics are independent of each other. This allows us to create an additional statistic by summing the individual PC statistics to create a combined statistic that follows a chi-squared distribution with additional degrees of freedom for each PC used.

Using the results from the PCA on the UK Biobank SNP set, we performed our selection scan on a different set of 516, 140 SNPs. We generated this set of SNPs by removing SNPs that were multi-allelic, had genotyping rates less than 99%, had minor allele frequencies less than 1%, and were not in Hardy-Weinberg equilibrium (*p* < 10^−6^).

We performed an allele frequency test for each novel SNP using the Nomenclature of Territorial Units for Statistics level 3 (NUTS3) classification of regions for the UK. The NUTS3 classification defines non-overlapping borders for each region in the UK, allowing us to uniquely map each individual to a region in the UK using their birth location coordinates by checking which NUTS3 regions they fell into. For each of our novel loci, we then performed an two-tailed *Z*-test between each region’s allele frequency against all other regions. We corrected for multiple testing using the Bonferroni correction.

## Supporting information

S1 Text Supplementary information. Section S1 contains additional information on the White British analysis. Section S2 compares our selection statistic to other existing selection statistics. Section S3 explores the time-scales of our selection hits. Section S4 explores the application of ProPCA to missing data. Section S5 details the implementation of ProPCA. Section S6 explains our variant of the Mailman algorithm for left multiplication. Section S7 details the convergence of ProPCA in the noiseless setting. Section S8 explores the contribution of the Mailman algorithm to scalability.
(PDF)

S1 Fig ProPCA is efficient at computing large numbers of PCs. Comparison of methods when calculating differing numbers of principal components. We computed principal components ranging from 1-40 on a dataset containing 20 populations separated at *F*_*st*_ = 0.01, 10, 000 individuals, and 50, 000 SNPs. All methods were run with default settings.
(PDF)

S2 Fig ProPCA has faster per-iteration runtimes versus FastPCA. Comparison of average per-iteration runtimes over simulated genotype data containing 100, 000 SNPs, six subpopulations, *F*_*st*_ = 0.10 and individuals varying from 10, 000 to 1, 000, 00. We were unable to leverage the source code for FlashPCA2 for this comparison.
(PDF)

S3 Fig ProPCA is computationally efficient relative to other methods. We compute the total time taken to estimate the top five principal components as a function of a measure of accuracy (MEV) for ProPCA compared to FastPCA and FlashPCA2. We performed these comparisons on simulated genotype data containing 50, 000 SNPs, 10, 000 individuals, six subpopulations, and *F*_*st*_ ∈ {0.001, 0.005, 0.01}.
(PDF)

S4 Fig ProPCA memory usage scales linearly. We display the memory usage in gigabytes of each method when computing the top 5 principal components. S4 Figa show the average memory usage from each method over 10 runs on a dataset containing six populations separated at *F*_*ST*_ = 0.01, 100, 000 SNPs, and individuals varying from 100,000 to 1,000,000. Figure S4 Figb shows a similar result, but with 100, 000 individuals and SNPs varying from 100,000 to 1,000,000 over a single run. All methods were run using default settings. We were unable to run bigstatsr for the SNPs experiment due to a bug that causes the method to crash in the presence of monomorphic SNPs.
(PDF)

S5 Fig ProPCA estimates principal components that are qualitatively indistinguishable from a full SVD on 1000 Genomes Phase 1 data. We applied our method to genotype data from Phase 1 of the 1000 Genomes project. On a dataset of 1, 092 individuals and 442, 350 SNPs, ProPCA computes the top five PCs in about 17 seconds on a single core. The top two PCs computed by ProPCA and by running SVD on this data set are qualitatively indistinguishable. EM refers to ProPCA.
(PDF)

S6 Fig Scatterplot pairs between the projections of the first five principal components of the unrelated White British. Plotting pairs of the first five principal components reveals structure amongst the unrelated White British. This structure diminishes as we increase the number principal components used.
(PDF)

S7 Fig Principal component scores of the unrelated White British overlaid on a map of the UK. Using birth location data available in the UK Biobank, we placed a scatter plot colored by principal component score to reveal geographic variation captured by the principal components.
(PDF)

S8 FigThe selection statistic is calibrated in the unrelated White British.We plot the theoretical quantiles of the $\chi_{1}^{2}$ distribution against each of the empirical quantiles observed from the first five principal components. All five principal components follow the theoretical distribution well until the upper tail. We additionally show the calibration of the combined statistic against the theoretical quantiles of the $\chi_{5}^{2}$ distribution.(PDF)

S9 Fig Boxplot of estimated allelic ages of putative signals of selection. Using allelic age estimates from the Human Genome Dating Atlas of Variant Age, we compared the estimated allelic ages of the significant signals of selection in Field et al. 2016 (SDS score > 4) and signals found by our own selection statistic. The *x*-axis denotes different clock models used to estimate allelic ages while allelic age estimates are denoted in generations on the *y*-axis. The joint clock model estimates allelic age using information from both the recombination and mutational clock models.
(PDF)

S10 Fig Proportion of total number of selection signal hits as a function of sample size. To further illustrate the importance of large sample sizes for biological discovery, we analyzed how many selection signals we could discover as a function of sample size. We randomly subsampled 10,000, 50,000, 100,000, and 200,000 individuals from the White British populations and performed our selection scan. The *x*-axis denotes sample size in thousands and the *y*-axis denotes the proportions of total hits discovered.
(PDF)

S11 Fig ProPCA infers more accurate principal components (PCs) in the presence of missing data compared to imputed genotypes. We compared the MEV from eigenvectors calculated from both modes of ProPCA with ground truth eigenvectors from performing a full SVD. We evaluated performance at 5% and 20% random missing values at 5 (S11a Fig) and 10 principal components (S11b Fig). We additionally compared ProPCA to mean imputation followed by a full SVD (S11c Fig). The data consists of simulated genotype data of 50, 000 SNPs from 10, 000 individuals from 5 populations for 5 PCs and 10 populations for 10 PCs separated by a range of *F*_*st*_ values. This process was repeated ten times to measure variability. Error bars denoting one standard deviation are shown for each point.
(PDF)

S12 Fig The Mailman matrix-vector multiplication contributes to the scalability of ProPCA. We compare the time taken to compute the top five principal components by the EM algorithm underlying ProPCA when used in conjunction with the Mailman algorithm and without. We performed these comparisons on simulated genotype data containing 100, 000 SNPs, six subpopulations, *F*_*st*_ = 0.10 and individuals varying from 10, 000 to 1, 000, 000. S12 Figa compares the runtime of the EM algorithm with the Mailman matrix-vector multiplication to an EM algorithm where the genotypes are represented as a matrix of doubles (EM1). With this representation, the EM algorithm could only be applied to sample sizes of at most 70, 000 individuals due to memory constraints. S12 Figb compares the runtime of the EM algorithm with the Mailman matrix-vector multiplication to an EM algorithm where genotypes are represented in a compact representation (EM2).
(PDF)

S1 Table Comparison of accuracy of methods to estimate principal components on the genotype data from the 1000 Genomes Phase 1 project. We compared the accuracy of the ProPCA algorithm, bigsnpr, FlashPCA2, PLINK2, TeraPCA, and FastPCA when applied to 1092 individuals in the 1000 Genomes Phase 1 project. We report MEV averaged over ten trials. FastPCA gave us a segmentation fault for estimation of ≥ 35 PCs. We ran all methods using default settings.
(PDF)

S2 Table Pearson correlation between principal components and birth location coordinates in the unrelated White British. Pearson correlation between the principal components and birth location coordinates reveals that the principal components unveil geographic variation. P-values from Pearson correlation *t*-test is shown in parentheses on the right.
(PDF)

S3 Table Selection statistics are not substantially inflated. We calculated the λ_*GC*_ values for each principal component and the combined statistic to check for inflation. In the unrelated White British set, the calculated values show that our selection statistics are not substantially inflated (top row). Furthermore, we show that the previously related statistic proposed by Galinsky et al. 2016 does not calibrate as well as our statistics based on λ_*GC*_ values (bottom row).
(PDF)

S4 Table Table of significant SNPs found by selection scan on unrelated White British. Our selection scan on the unrelated White British population resulted in 59 significant SNPs. Our significance threshold was a Bonferroni corrected *p* < 0.05. Bonferroni corrected *p*-values are shown.
(XLSX)

S5 Table Principal component selection scan reveals 12 unique loci under selection across the top five principal components. We obtained 59 selection hits across the first five principal components of the unrelated White British subset of the UK Biobank. We clustered these hits into 12 unique loci by aggregating all significant hits into 1 Mb windows centered around the most significant hits. Other genes with significant hits that are within the 1 Mb window are listed in the last column.
(PDF)

S6 Table Allele frequency tests between NUTS3 regions at novel loci confirms differences between geographic regions. We performed a two-tailed proportion test for our novel loci between the allele frequency in each individual region from the NUTS3 classification of the United Kingdom against the frequency from every other region. We corrected the *p*-values using the Bonferroni correction (11 loci × 163 regions). The corrected *p*-values for regions passing the significance threshold are shown in the table.
(PDF)

S7 Table Combined selection statistic across the top five principal components reveals four additional novel loci. We discover four additional novel loci using our combined selection statistic from the first five principal components. Loci not found in the individual PC selection statistics are denoted by an asterik in the rsid column. The chi-squared statistic (one degree of freedom) for each principal component is shown in the last five columns of the table.
(PDF)

S8 Table Selection hits are associated with phenotypes in the UK Biobank. We ran genome-wide association tests for 64 phenotypes in the full release of the UK Biobank for each of our loci. Phenotypes shown reached a *p*-value of genome-wide significance level (0.05 × 10^−6^).
(PDF)

S9 Table Selection hits are associated with phenotypes from the Global Biobank Engine. We queried the Global Biobank Engine for associations from our loci. The Global Biobank Engine contains GWAS results for many more phenotypes than those available in the UK Biobank. Phenotypes shown are significant at genome-wide significance level (0.05 × 10^−6^).
(PDF)

## References

[1] Novembre J, Ramachandran S. Perspectives on human population structure at the cusp of the sequencing era. Annual review of genomics and human genetics. 2011;12:245–274. 21801023 10.1146/annurev-genom-090810-183123

[2] Novembre J, Johnson T, Bryc K, Kutalik Z, Boyko A, Auton A, et al Genes mirror geography within Europe. Nature. 2008;456(7219):274. 10.1038/nature07331 PMC2735096 18758442

[3] Yang WY, Novembre J, Eskin E, Halperin E. A model-based approach for analysis of spatial structure in genetic data. Nature genetics. 2012;44(6):725–731. 10.1038/ng.2285 22610118 PMC3592563

[4] Baran Y, Quintela I, Carracedo Á, Pasaniuc B, Halperin E. Enhanced localization of genetic samples through linkage-disequilibrium correction. The American Journal of Human Genetics. 2013;92(6):882–894. 10.1016/j.ajhg.2013.04.023 23726367 PMC3675263

[5] Price AL, Zaitlen NA, Reich D, Patterson N. New approaches to population stratification in genome-wide association studies. Nature reviews Genetics. 2010;11(7):459 10.1038/nrg2813 20548291 PMC2975875

[6] Patterson N, Price AL, Reich D. Population Structure and Eigenanalysis. PLoS Genetics. 2006;2(12):e190+. 10.1371/journal.pgen.0020190 17194218 PMC1713260

[7] Hanis CL, Chakraborty R, Ferrell RE, Schull WJ. Individual admixture estimates: disease associations and individual risk of diabetes and gallbladder disease among Mexican-Americans in Starr County, Texas. Am J Phys Anthropol. 1986;70(4):433–441. 3766713 10.1002/ajpa.1330700404

[8] Pritchard J, Stephens M, Donnelly P. Inference of Population Structure Using Multilocus Genotype Data. Genetics. 2000;155:945–959. 10835412 10.1093/genetics/155.2.945 PMC1461096

[9] Chen C, Durand E, Forbes F, François O. Bayesian clustering algorithms ascertaining spatial population structure: a new computer program and a comparison study. Molecular Ecology Resources. 2007;7(5):747–756.

[10] Engelhardt BE, Stephens M. Analysis of population structure: a unifying framework and novel methods based on sparse factor analysis. PLoS genetics. 2010;6(9):e1001117 10.1371/journal.pgen.1001117 20862358 PMC2940725

[11] Jolliffe IT. Principal Component Analysis and Factor Analysis In: Principal component analysis. Springer; 1986 p. 115–128.

[12] Galinsky KJ, Bhatia G, Loh PR, Georgiev S, Mukherjee S, Patterson NJ, et al Fast principal-component analysis reveals convergent evolution of ADH1B in Europe and East Asia. The American Journal of Human Genetics. 2016;98(3):456–472. 10.1016/j.ajhg.2015.12.022 26924531 PMC4827102

[13] Abraham G, Qiu Y, Inouye M. FlashPCA2: principal component analysis of biobank-scale genotype datasets. Bioinformatics. 2017;. 28475694 10.1093/bioinformatics/btx299

[14] Prive F, Aschard H, Ziyatdinov A, Blum M. Efficient analysis of large-scale genome-wide data with two R packages: bigstatsr and bigsnpr. Bioinformatics. 2018;34(16):2781–2787. 10.1093/bioinformatics/bty185 29617937 PMC6084588

[15] Bose A, Kalantzis V, Kontopoulou EM, Elkady M, Paschou P, Drineas P. TeraPCA: a fast and scalable software package to study genetic variation in tera-scale genotypes. Bioinformatics. 2019;35(19):3679–3683. 30957838 10.1093/bioinformatics/btz157

[16] Chang C, Chow C, Tellier L, Vattikuti S, Purcell S, Lee J. Second-generation PLINK: rising to the challenge of larger and richer datasets. Gigascience. 2015;4:7 10.1186/s13742-015-0047-8 25722852 PMC4342193

[17] Price A, Patterson N, Plenge R, Weinblatt M, Shadick N, Reich D. Principal components analysis corrects for stratification in genome-wide association studies. Nature Genetics. 2006;38:904–909. 16862161 10.1038/ng1847

[18] Canela-Xandri O, Law A, Gray A, Woolliams JA, Tenesa A. A new tool called DISSECT for analysing large genomic data sets using a Big Data approach. Nature communications. 2015;6:10162 10.1038/ncomms10162 26657010 PMC4682108

[19] Roweis ST. EM algorithms for PCA and SPCA In: Advances in neural information processing systems; 1998 p. 626–632.

[20] Tipping ME, Bishop CM. Probabilistic principal component analysis. Journal of the Royal Statistical Society: Series B (Statistical Methodology). 1999;61(3):611–622.

[21] Liberty E, Zucker SW. The mailman algorithm: A note on matrix–vector multiplication. Information Processing Letters. 2009;109(3):179–182.

[22] Bycroft C, Freeman C, Petkova D, Band G, Elliott LT, Sharp K, et al The UK Biobank resource with deep phenotyping and genomic data. Nature. 2018;562:203–209. 10.1038/s41586-018-0579-z 30305743 PMC6786975

[23] Purcell S, Neale B, Todd-Brown K, Thomas L, Ferreira MA, Bender D, et al PLINK: a tool set for whole-genome association and population-based linkage analyses. The American Journal of Human Genetics. 2007;81(3):559–575. 10.1086/519795 17701901 PMC1950838

[24] The 1000 Genomes Project Consortium. An integrated map of genetic variation from 1,092 human genomes. Nature. 2012;491(7422):56–65. 23128226 10.1038/nature11632 PMC3498066

[25] Shriver MD, Kennedy GC, Parra EJ, Lawson HA, Sonpar V, Huang J, et al The genomic distribution of population substructure in four populations using 8,525 autosomal SNPs. Human genomics. 2004;1(4):274 10.1186/1479-7364-1-4-274 15588487 PMC3525267

[26] Tian C, Plenge RM, Ransom M, Lee A, Villoslada P, Selmi C, et al Analysis and application of European genetic substructure using 300 K SNP information. PLoS genetics. 2008;4(1):e4 10.1371/journal.pgen.0040004 18208329 PMC2211544

[27] Wiegering A, Ruther U, Gerhardt C. The ciliary protein Rpgrip1l in development and disease. Dev Biol. 2018;442(1):60–68. 30075108 10.1016/j.ydbio.2018.07.024

[28] Delous M, Baala L, Salomon R, Laclef C, Vierkotten J, Tory K, et al The ciliary gene RPGRIP1L is mutated in cerebello-oculo-renal syndrome (Joubert syndrome type B) and Meckel syndrome. Nature Genetics. 2007;39:875–881. 17558409 10.1038/ng2039

[29] Devuyst O, Arnould VJ. Mutations in RPGRIP1L: extending the clinical spectrum of ciliopathies. Nephrology Dialysis Transplantation. 2008;23(5):1500–1503. 10.1093/ndt/gfn033 18281315

[30] Khanna H, Davis EE, Murga-Zamalloa CA, Estrada-Cuzcano A, Lopez I, den Hollander AI, et al A common allele in RPGRIP1L is a modifier of retinal degeneration in ciliopathies. Nature Genetics. 2009;41(6):739–45. 10.1038/ng.366 19430481 PMC2783476

[31] Aschard H, Vilhjálmsson B, Greliche N, Morange P, Trégouët D, Kraft P. Maximizing the power of principal-component analysis of correlated phenotypes in genome-wide association studies. AJHG. 2014;94(5):662–76. 10.1016/j.ajhg.2014.03.016 PMC4067564 24746957

[32] Korneev K, Atretkhany K, Drutskaya M, Grivennikov S, Kuprash D, Nedospasov S. TLR-signaling and proinflammatory cytokines as drivers of tumorigenesis. Cytokine. 2017;89:127–135. 26854213 10.1016/j.cyto.2016.01.021

[33] Mockenhaupt F, Cramer J, Hamann L, Stegemann M, Eckert J, Oh N, et al Toll-like receptor (TLR) polymorphisms in African children: Common TLR-4 variants predispose to severe malaria. PNAS. 2006;103(1):177–182. 10.1073/pnas.0506803102 16371473 PMC1324982

[34] Van der Graaf C, Netea M, Morré S, Den Heijer M, Verweij P, Van der Meer J, et al Toll-like receptor 4 Asp299Gly/Thr399Ile polymorphisms are a risk factor for Candida bloodstream infection. European Cytokine Network. 2006;17(1):29–34. 16613760

[35] Field Y, Boyle EA, Telis N, Gao Z, Gaulton KJ, Golan D, et al Detection of human adaptation during the past 2000 years. Science. 2016;354(6313):760–764. 10.1126/science.aag0776 27738015 PMC5182071

[36] Albers, McVean. Dating genomic variants and shared ancestry in population-scale sequencing data. bioRxiv. 2019. 10.1371/journal.pbio.3000586 PMC6992231 31951611

[37] Wu Y, Sankararaman S. A scalable estimator of SNP heritability for biobank-scale data. Bioinformatics. 2018;34(13):i187–i194. 10.1093/bioinformatics/bty253 29950019 PMC6022682

[38] Halko N, Martinsson PG, Tropp JA. Finding structure with randomness: Probabilistic algorithms for constructing approximate matrix decompositions. SIAM review. 2011;53(2):217–288.

[39] Mathieson I, McVean G. Differential confounding of rare and common variants in spatially structured populations. Nature genetics. 2012;44(3):243 10.1038/ng.1074 22306651 PMC3303124

[40] Hellenthal G, Auton A, Falush D. Inferring Human Colonization History Using a Copying Model. PLoS Genet. 2008;4(5):e1000078 10.1371/journal.pgen.1000078 18497854 PMC2367454

[41] Li N, Stephens M. Modeling Linkage Disequilibrium and Identifying Recombination Hotspots Using Single-Nucleotide Polymorphism Data. Genetics. 2003;165(4):2213–2233. 14704198 10.1093/genetics/165.4.2213 PMC1462870

[42] Wen X, Stephens M. Using linear predictors to impute allele frequencies from summary or pooled genotype data. The annals of applied statistics. 2010;4(3):1158 21479081 10.1214/10-aoas338 PMC3072818

[43] Schein AI, Saul LK, Ungar LH. A generalized linear model for principal component analysis of binary data. In: AISTATS. vol. 3; 2003. p. 10.

[44] Li W, Cerise J, Yang Y, Han H. Application of t-SNE to human genetic data. J Bioinform Comput Biol. 2017;15(4):1750017 28718343 10.1142/S0219720017500172

[45] Becht E, McInnes L, Healy J, Dutertre C, Kwok I, Ng L, et al Dimensionality reduction for visualizing single-cell data using UMAP. Nat Biotechnol. 2019;37:38–44. 10.1038/nbt.4314 30531897

[46] Anderson TW, Rubin H. Statistical inference in factor analysis. In: Proceedings of the third Berkeley symposium on mathematical statistics and probability. vol. 5; 1956. p. 111–150.

[47] Szlam A, Tulloch A, Tygert M. Accurate Low-Rank Approximations Via a Few Iterations of Alternating Least Squares. SIAM Journal on Matrix Analysis and Applications. 2017;38(2):425–433.

[48] Lehoucq RB, Sorensen DC. Deflation techniques for an implicitly restarted Arnoldi iteration. SIAM Journal on Matrix Analysis and Applications. 1996;17(4):789–821.

[49] Manichaikul A, Mychaleckyj J, Rich S, Daly K, Sale M, Chen W. Robust relationship inference in genome-wide association studies. Bioinformatics. 2010;26(22):2867–2873. 10.1093/bioinformatics/btq559 20926424 PMC3025716

