# Supplementary material for: Scalable probabilistic PCA for large-scale genetic variation data
Source: PLoS Genet. 2020 May 29;16(5):e1008773. doi: 10.1371/journal.pgen.1008773 (PMC7286535; doi:10.1371/journal.pgen.1008773)
Supplement: S1 Text — Section S1 contains additional information on the White British analysis. Section S2 compares our selection statistic to other existing selection statistics. Section S3 explores the time-scales of our selection hits. Section S4 explores the application of ProPCA to missing data. Section S5 details the implementation of ProPCA. Section S6 explains our variant of the Mailman algorithm for left multiplication. Section S7 details the convergence of ProPCA in the noiseless setting. Section S8 explores the contribution of the Mailman algorithm to scalability. (PDF) [file pgen.1008773.s001.pdf]

Supplementary Information: Scalable Probabilistic PCA for large-scale genetic variation data

Contents

S1 White British Selection Scan and Analysis 1

S2 Comparison of Selection Statistics 2

S3 Time-scale of selection hits 2

S4 Application of ProPCA to missing data 3
S4.1 PCA with Missing Data 3
S4.2 EM for PCA with Missing Data 3

S5 Implementation details 4

S6 Novel variant of the Mailman algorithm for left multiplication 5

S7 Convergence of ProPCA in the noiseless setting 6

S8 Exploring the contribution of the Mailman algorithm to scalability 6

S1 White British Selection Scan and Analysis

Among the significant loci that we did not highlight in the main text, there are several genic loci have biological significance.

Transcriptome-wide association studies (TWAS) suggest that gene expression at *HERC6* is associated with gout ($p = 3.8 \times 10^{-123}$) [1]. Epidemiological studies in the UK also have shown that Wales, the geographic region associated with differences in *HERC6* allele frequencies, is among the regions of the UK with the highest prevalence and incidence in the UK [2]. The specific variant that is putatively under selection at this locus, rs112873858, does not appear to be significantly associated with gout in the UK Biobank however (logistic regression $p = 0.2395$).

HERC2 (hect domain and RLD2), contains a single SNP in *HERC2* that is a primary determinant of light eye color in modern Europeans [3] and has been previously shown to be under selection [4]. A number of other SNPs in the *HERC2* locus have also been shown to be associated with iris color [5]. In the UK Biobank, we find that the SNP with the most significant p-value in *HERC2*, rs1129038, is associated with childhood sunburn occasions ($p = 6 \times 10^{-134}$) as well as skin and hair pigmentation ($p = 9.4 \times 10^{-103}$) (Table S8,S9).

SKI is a proto-oncogene located at a region close to the p73 tumor suppressor gene [6]. It is implicated in the TGF-β signaling pathway [7] and has been shown to play a role in a variety of

cancers [6, 8]. However, our specific locus does not appear to be significantly associated with any cancer in the UK Biobank.

Our combined selection statistic also resulted in an additional genic loci we did not highlight in the main text. The *AMPH* locus is located in the gene that codes for the amphiphysin protein, which is associated with the cytoplasmic surface of synaptic vesicles [9]. The gene is also implicated in stiff person syndrome and breast cancer [9]; however we were unable to find any significant associations with traits in the UK Biobank.

S2 Comparison of Selection Statistics

Several approaches have been previously proposed [10–12] to discovering signals of putative selection based on PCA. These approaches look for variants with large differences in alleles frequencies between populations or individuals differentiated along an axis (principal component). Typically, the PCs correspond to population structure so that these methods correspond to tests for SNPs that are not well described by the PCs. The proposed statistics attempt to detect SNPs as outliers relative to the structure captured by either a single PC or the space spanned by k PCs. The differences across all these statistics arise from the statistical assumptions of the underlying model of population structure.

[11] examines several statistics to rank SNPs based on the PC loadings and uses an outlier approach to determine putative targets of selection. [10] formulates a hypothesis testing framework to show that, under a model of drift, their proposed statistic for the k-th PC has a chi-squared distribution with one degree of freedom. [12] employs a chi-squared Malahanobis distance distance as a means of outlier detection after regressing each SNP by the k principal components.

Our proposed statistic is similar to the statistic proposed in [12] in its use of an outlier detection approach, *i.e.*, looking for SNPs that are not well-described by the first K PCs. To aid interpretability, we further project the residual variance along each of the k PCs, in turn, to identify the specific axes of variation along which the SNP tends to be an outlier.

S3 Time-scale of selection hits

To better understand the time-scale of the episode of selection that our proposed statistic is sensitive to, we examined the estimated allelic ages of the mutations at the hits detected by our statistic. We obtained estimates of allelic ages using the Human Genome Dating Atlas of Variant Age [13]. We restricted our analysis to ages estimated from variants genotyped in the 1000 Genomes Project [14]. Further, the underlying method for estimating variant ages assumes that the alternate allele is the derived allele. When this assumption is violated, the resulting estimates may not be valid. Thus, we restricted our analysis to variants at which the alternate allele is the derived allele to obtain a total of 42 variants (out of our initial list of 63 hits that are significant across each of the five PCs as well as the combined statistic). The mean ages of these alleles was estimated to be around $11,555$ generations using the mutation clock, $18,946$ generations using the recombination clock, and $19,007$ generations using the combined clock. However, there is substantial variation in the allelic ages estimates. For eample, 17 of the variants have ages less than $5,000$ generations using the combined clock.

We compared our allelic ages estimates to those of the hits from a recent work designed to detect recent episodes of positive selection [15]. We restricted our analysis to the list of variants from the UK10K with SDS scores > 4. This resulted in 1620 variants out of a total of $4,451,435$ variants with SDS scores available (top 4×10^{-4} of the SDS scores). We then used the allelic

ages for each of these variants available from the Human Genome Dating Atlas again restricting our analysis to those variants where the alternate allele matches the derived allele yielding a list of 920 variants. The mean ages for these variants are approximately 7,620 generations (mutation clock), 12,471 generations (recombination clock), and 11,944 generations (combined clock). We note that there is considerable variation in the ages across variants. Figure S9 shows that the variants identified by the SDS statistic tend to younger on average than those from our statistic (mean age of 12,471 generations for SDS vs 18,700 for our statistic). This difference is nominally statistically significant using a Mann-Whitney-Wilcoxon test ($p = 0.002$,0.001, and 8×10^{-4} for each of the mutation, recombination, and combined clocks). We caution however that the hits in each of the lists are unlikely to be statistically independent (for example, there are multiple variants that are present in the LCT locus). Further, there is considerable uncertainty associated with the age of these variants and a more careful analysis would need to account for this uncertainty.

S4 Application of ProPCA to missing data

S4.1 PCA with Missing Data

The use of a probabilistic model allows for handling missing entries in the genotype matrix. We assume that the genotype data is missing at random (MAR) [16], *i.e.*, the missingness depends only on the other observed values. We partition the observed data $\boldsymbol{G}$ into observed and unobserved entries. In the missing data setting, the observation model becomes:

$$\boldsymbol{g}_i|\boldsymbol{x}_i, \boldsymbol{\epsilon}_i = \boldsymbol{\mu} + \boldsymbol{C}\boldsymbol{x}_i + \boldsymbol{\epsilon}_i \quad (1)$$

Here $\boldsymbol{\mu}$ is a length m vector denoting the mean genotype vector. Unlike the fully observed setting where the maximum likelihood estimate of $\boldsymbol{\mu}$ is equal to the sample mean $\overline{\boldsymbol{g}}$, in the missing data setting, we need to estimate $\boldsymbol{\mu}$ within the EM algorithm.

$$\begin{aligned} O &= \{\ (i,j) \ \mid \ g_{ij} \ is\ observed \ \}, \\ O_j &= \{\ i \ \mid \ (i,j) \in O \ \}, \\ O_i &= \{\ j \ \mid \ (i,j) \in O \ \}, \\ \boldsymbol{x}_j &= j^{th}\ column\ of\ \mathbf{X}, \\ \boldsymbol{c}_i &= i^{th}\ row\ of\ \mathbf{C}\ written\ as\ a\ column, \\ \mu_i &= the\ mean\ of\ g_{ij}\ where\ j \in O_i \end{aligned}$$

S4.2 EM for PCA with Missing Data

$$\text{E Step:} \qquad \boldsymbol{x}_j = (\sum_{i \in O_j} \boldsymbol{c}_i \boldsymbol{c}_i^T)^{-1} \sum_{i \in O_j} \boldsymbol{c}_i (y_{ij} - \mu_i) \quad (2)$$

$$\text{M Step:} \qquad \boldsymbol{c}_i = (\sum_{j \in O_i} \boldsymbol{x}_j \boldsymbol{x}_j^T)^{-1} \sum_{j \in O_i} (y_{ij} - \mu_i) \boldsymbol{x}_j \quad (3)$$

$$\mu_i = \frac{1}{|O_i|} \sum_{j \in O_i} (g_{ij} - \boldsymbol{c}_i^T \boldsymbol{x}_j) \quad (4)$$

Using the same ideas of the Mailman algorithm, the EM algorithm for missing data has a running time of $\mathcal{O}(\frac{nmk}{max(\log_3 n, \log_3 m)} + n_{missing} k^2)$ per iteration. Since the percentage of missing data is quite low, we can use the probabilistic model to efficiently handle missing data.

We evaluated the effectiveness of this extended model using simulated genotypes with missing data (Figure S11). We compared the accuracy of the PCs estimated using the extended model to the PCs estimated by running the EM algorithm on genotype data that was imputed through a random draw from a binomial distribution parameterized by the allele frequencies.

We simulated ten sets of complete genotypes with 50,000 SNPs and 10,000 individuals from 5 and 10 populations, each at differing F_{ST} levels from 0.001 to 0.01 at intervals of 0.001. We simulated missing data by randomly removing 5% and 20% of the genotypes. To estimate the variance of our method, we averaged over 10 datasets.

For each method tested, we computed the MEV between the PCs inferred from the missing data and the PCs computed by applying SVD to the original genotype data with no missing values. Figure S11 shows that the PCs inferred from the ProPCA implementation that explicitly handles missing data are more accurate than the PCs computed by running ProPCA on imputed genotypes (Figure S11a, S11b). Furthermore, we see that ProPCA can infer PCs comparable to running mean imputation followed by a full SVD (Figure S11c).

S5 Implementation details

Application of the Mailman algorithm to the EM algorithm For a genotype matrix $\boldsymbol{G}$ where $m > \lceil \log_3(n) \rceil$, we partition $\boldsymbol{G} = \left(\boldsymbol{G}_1^{\mathrm{T}} \ldots \boldsymbol{G}_B^{\mathrm{T}}\right)^{\mathrm{T}}$ into $B = \lceil \frac{m}{log_3(n)} \rceil$ sub-matrices each of size $\lceil log_3(n) \times n \rceil$ and decompose each $\boldsymbol{G}_b = \boldsymbol{U}_n \boldsymbol{P}_b$.

The M-step (Equation 5) requires computing $\boldsymbol{G\alpha}$ for k distinct vectors $\boldsymbol{\alpha}$. We compute $\boldsymbol{G\alpha} = \begin{pmatrix} G_1\boldsymbol{\alpha} \\ G_2\boldsymbol{\alpha} \\ \vdots \\ G_B\boldsymbol{\alpha} \end{pmatrix}$. Since each of the products $\boldsymbol{G}_b\boldsymbol{\alpha}, b \in \{1, \ldots, B\}$ can be computed in $\mathcal{O}(n)$ operations (given $\boldsymbol{U}_n$, and $\boldsymbol{P}_b$), the entire matrix-vector product $\boldsymbol{G\alpha}$ can be computed in $\mathcal{O}(\frac{nm}{\log_3(n)})$ time.

The E-step (Equation 4) requires computing $\boldsymbol{\beta}^{\mathrm{T}}\boldsymbol{G}$ for k distinct vectors $\boldsymbol{\beta}$. We compute this product as $\sum_{b=1}^{B} \boldsymbol{\beta}_b^{\mathrm{T}} \boldsymbol{G}_b$ in $\mathcal{O}(\frac{nm}{\log_3(n)})$ time where each term in the sum is computed using our novel variant of the Mailman algorithm.

Likelihood Computation To check for convergence, we need to compute the likelihood of the parameters in each iteration of the EM algorithm which is equivalent to the computing the squared Frobenius norm of the error matrix, *i.e.*, $||\mathbf{Y} - \mathbf{CX}||_F^2$.

$$\begin{aligned} ||\mathbf{Y} - \mathbf{CX}||_F^2 &= tr[\ (\mathbf{Y} - \mathbf{CX})(\mathbf{Y} - \mathbf{CX})^T] \\ &= -2tr(\mathbf{Y^T C X}) + tr(\mathbf{X^T C^T C X}) + const \end{aligned}$$

Let $\boldsymbol{Z} = \boldsymbol{C}^{\mathrm{T}}\boldsymbol{Y}$. $\boldsymbol{Z}$ and $\boldsymbol{X}$ are $k \times n$ matrices so that the first term in the sum above ($tr(\boldsymbol{Z}^{\mathrm{T}}\boldsymbol{X})$) can be computed in $\mathcal{O}(nk)$ time. $\boldsymbol{Z}$ can be computed in $\mathcal{O}(\frac{nmk}{max(\log_3(n), \log_3(m)})$ using the Mailman algorithm. Thus, the likelihood can be computed in $\mathcal{O}(\frac{nmk}{max(\log_3(n), \log_3(m)} + nk)$.

We note that the columns of the Maximum Likelihood Estimate (MLE) of $\mathbf{C}$ do not correspond to the principal components of $\boldsymbol{Y}$ but instead span the principal subspace of the top k eigenvectors of $\mathbf{Y}$. We can orthogonalize the matrix $\mathbf{C}$ to obtain the principal components in time $\mathcal{O}(mk^2)$, using *e.g.*, the Q-R decomposition.

Efficient implementation of the Mailman algorithm There are several considerations in an efficient practical implementation of the Mailman algorithm. While the multiplication with the $\boldsymbol{U}$ matrix is obtained by a recursion, we convert this into an iterative algorithm. Another important factor arises from the fact that the Mailman algorithm needs access to elements in the input vector that are not necessarily located in consecutive memory addresses. This can lead to frequent cache misses that can substantially reduce the efficiency of the implementation. To get around this limitation, we implemented a batched version of the Mailman algorithm. This version uses the idea that typically we need to multiply more than one vector at a time, *e.g.*, we often need to compute $k = 5$ PCs. Our implementation operates on the batch of input vectors at a time using the resulting locality among the input vectors. We use a default batch size of 10 although other batch sizes could also be used.

Memory considerations In the mailman algorithm, the matrix $\boldsymbol{U}_n$ is only used implicitly and need not be stored. The $\boldsymbol{P}$ matrix has the property that each column has exactly one entry that is one while all the other entries are zero. $\boldsymbol{P}$ can be stored as a length n vector $\boldsymbol{p}$ indicating the locations of the one entry in each column of $\boldsymbol{P}$. Since each element of the $\boldsymbol{p}$ vector is an integer, such that $p_i \in [1, n], i \in \{1, \ldots, n\}$, we can store $\boldsymbol{p}$ in $\lceil log_2(n) \rceil$ bits. This can be efficiently represented by storing 2 or more elements of p in a single four byte integer. The storing and retrieval of an element can be performed by bit operations which increase the computational complexity moderately while reducing the memory requirements considerably.

S6 Novel variant of the Mailman algorithm for left multiplication

The EM algorithm requires alternate left and right multiplication of genotype matrix $\boldsymbol{G}$ in the E- and M-steps respectively. One approach to using the Mailman algorithm for each step consists of partitioning $\boldsymbol{G}$ along the columns and the rows respectively followed by computing decompositions of each of the resulting sub-matrices. This approach, however, doubles the memory requirement of the resulting algorithm. Instead, we propose a variant of the Mailman algorithm for left multiplication of a matrix with a vector that uses the same decomposition as for right multiplication.

Recall that for right multiplication, we would like to compute $\boldsymbol{c} = \boldsymbol{A}\boldsymbol{b}$ for an arbitrary real-valued vector $\boldsymbol{b}$ and a $m \times n$ matrix $\boldsymbol{A}$ whose entries take values in $\{0, 1, 2\}$. We assume that $m = \lceil \log_3(n) \rceil$. The Mailman algorithm decomposes $\boldsymbol{A}$ as $\boldsymbol{A} = \boldsymbol{U}_m\boldsymbol{P}$. Here $\boldsymbol{U}_m$ is the $m \times m_0$ matrix whose columns containing all $m_0 = 3^m$ possible vectors over $\{0, 1, 2\}$ of length m. $\boldsymbol{P}$ is a $m_0 \times n$ matrix. We set an entry $P_{i,j}$ to 1 if column j of $\boldsymbol{A}$ matches column i of U_m: $A^{(j)} = U_m^{(i)}$. All other entries of $\boldsymbol{P}$ are set to zero. The decomposition of any matrix $\boldsymbol{A}$ into $\boldsymbol{U}_m$ and $\boldsymbol{P}$ can be done in $\mathcal{O}(nm)$ time. Given this decomposition, the desired product $\boldsymbol{c}$ is computed in two steps, each of which has $\mathcal{O}(n)$ time complexity [17]:

$$\boldsymbol{d} = \boldsymbol{P}\boldsymbol{b}, \quad \boldsymbol{c} = \boldsymbol{U}_m\boldsymbol{d}$$

We now describe an algorithm to compute $\boldsymbol{f}^{\mathrm{T}} = \boldsymbol{e}^{\mathrm{T}}\boldsymbol{A}$ using the same decomposition $\boldsymbol{A} = \boldsymbol{U}_m\boldsymbol{P}$. As in the setting of right multiplication, this algorithm proceeds in two steps:

$$\boldsymbol{g}^{\mathrm{T}} = \boldsymbol{e}^{\mathrm{T}}\boldsymbol{U}_m, \quad \boldsymbol{f}^{\mathrm{T}} = \boldsymbol{g}^{\mathrm{T}}\boldsymbol{P}$$

For the first step, we have:

$$\begin{aligned}
\boldsymbol{g}^{\mathrm{T}} = \boldsymbol{e}^{\mathrm{T}}\boldsymbol{U}_m &= \begin{pmatrix} e_1 & \boldsymbol{e}_{2:m}^{\mathrm{T}} \end{pmatrix} \begin{pmatrix} \boldsymbol{0}_{3^{m-1}}^{\mathrm{T}} & \boldsymbol{1}_{3^{m-1}}^{\mathrm{T}} & \boldsymbol{2}_{3^{m-1}}^{\mathrm{T}} \\ \boldsymbol{U}_{m-1} & \boldsymbol{U}_{m-1} & \boldsymbol{U}_{m-1} \end{pmatrix} \\
&= \begin{pmatrix} \boldsymbol{e}_{2:m}^{\mathrm{T}}\boldsymbol{U}_{m-1} & e_1\boldsymbol{1}_{3^{m-1}} + \boldsymbol{e}_{2:m}^{\mathrm{T}}\boldsymbol{U}_{m-1} & e_1\boldsymbol{2}_{3^{m-1}}^{\mathrm{T}} + \boldsymbol{e}_{2:m}^{\mathrm{T}}\boldsymbol{U}_{m-1} \end{pmatrix} \qquad (5)
\end{aligned}$$

Here e_1 is the first element of $\boldsymbol{e}$ and $\boldsymbol{e}^{\mathrm{T}}_{2:m}$ is a vector of length $m-1$ consisting of elements 2 to m of vector $\boldsymbol{e}$.

This gives us a recursive algorithm to compute $\boldsymbol{g}$ with base case :

$$\begin{aligned} e_m \boldsymbol{U}_1 &= e_m \begin{pmatrix} 0 & 1 & 2 \end{pmatrix} \\ &= \begin{pmatrix} 0 & e_m & 2e_m \end{pmatrix} \end{aligned} \tag{6}$$

The time complexity of this algorithm is given by $T(m) \leq 3^m + T(m-1) \leq 3^{m+1} = 3 \times 3^{\lceil \log_3(n) \rceil} = \mathcal{O}(n)$.

For the second step, note that each column of $\boldsymbol{P}$ has exactly one non-zero entry (with value equal to one). Thus, each entry of $\boldsymbol{f}$ can be computed in constant time so that $\boldsymbol{f}$ can be computed in $\mathcal{O}(3^m) = \mathcal{O}(n)$ time.

Thus, the total time complexity of computing $\boldsymbol{f}$ is $\mathcal{O}(n)$ instead of $\mathcal{O}(n \log_3(n))$ using naive matrix-vector multiplication.

For a general matrix $\boldsymbol{A}$ where $m > \lceil \log_3(n) \rceil$, we partition $\boldsymbol{A} = \begin{pmatrix} \boldsymbol{A}_1 \\ \boldsymbol{A}_2 \\ \vdots \\ \boldsymbol{A}_B \end{pmatrix}$ into $B = \lceil \frac{m}{log_3(n)} \rceil$ sub-matrices each of size $\lceil log_3(n) \times n \rceil$ and decompose each $\boldsymbol{A}_b = \boldsymbol{U}_n \boldsymbol{P}_b$. To now compute $\boldsymbol{f}^{\mathrm{T}} = \boldsymbol{e}^{\mathrm{T}} \boldsymbol{A}$, we compute $\sum_{b=1}^{B} \boldsymbol{e}_b^{\mathrm{T}} \boldsymbol{A}_b$. Each product can be computed in $\mathcal{O}(n)$ time so that $\boldsymbol{f}$ can be computed in $\mathcal{O}(\frac{nm}{\log_3(n)})$.

S7 Convergence of ProPCA in the noiseless setting

There are several techniques to analyze the convergence properties of ProPCA. Under the assumption that the linear Gaussian model is true, convergence results of the EM algorithm can be invoked [18]. An alternate view of convergence in the setting where $\sigma^2 \to 0$ arises from viewing the EM updates as mathematically equivalent to alternating least squares [19]. In this view, we can show that the spectral norm of the reconstruction error, *i.e.*, the error between the data matrix $\boldsymbol{Y}$ and its rank-k approximation $\boldsymbol{CX}$, decreases to the optimal value at a rate that is exponential in the number of iterations. Our arguments follow from a combination of previous theoretical results.

The range of the matrix $\boldsymbol{C}^{(t)}$ obtained at the end of iteration t of the EM algorithm is the same as the range of the matrix $\boldsymbol{Y}\boldsymbol{Y}^{\mathrm{T}^t}\boldsymbol{C}_0$ (Theorem 5 of Szlam et al. 2017). Setting $\boldsymbol{C}_0 = \boldsymbol{Y}\boldsymbol{\Omega}$ where $\boldsymbol{\Omega}$ where $\boldsymbol{\Omega}$ is a $n \times l$ matrix ($l = 2k$) with entries drawn independently from a standard normal distribution. Let $\boldsymbol{Q}^{(t)}$ denote the orthonormal basis for the range of $\boldsymbol{C}^{(t)}$. Then $\mathbb{E}\left[\|\boldsymbol{Y} - \boldsymbol{Q}^{(t)}{\boldsymbol{Q}^{(t)}}^T\boldsymbol{Y}\right]\| \leq (1+\alpha)^{\frac{1}{2t+1}}\sigma_{k+1}$ (Corollary 10.10 of Halko *et al.*, 2009). Here σ_{k+1} is the $(k+1)^{st}$ largest singular value of $\boldsymbol{Y}$ and α is a constant that depends on the m, n and k.

S8 Exploring the contribution of the Mailman algorithm to scalability

To explore the contribution of the Mailman algorithm to the scalability, we explored variants of the EM algorithm underlying ProPCA that differ in the implementation of the core genotype matrix-vector multiplication. In addition to the Mailman algorithm for genotype matrix-vector multiplication (EM-Mailman), we considered an implementation where the genotypes are stored as a matrix of doubles using the Eigen matrix library [20] (EM1) as well as another implementation

where the genotypes are stored in a compact representation in which each genotype is represented using two bits (EM2). The representation in EM2 is expected to be memory-efficient relative to EM1. However, since EM1 represents genotypes directly as a matrix object in Eigen, we expect EM1 to be computationally more efficient. Figure S12 supports this intuition. EM1 could only be applied to sample sizes of up to $70,000$ before reaching our memory limit. While EM2 can run sample sizes up to $1,000,000$, it is more than two orders of magnitude slower than EM-Mailman. While EM1 is substantially faster, EM-Mailman is about three times faster. We expect that, even if memory were not a constraint, the Mailman algorithm would remain faster than the basic EM algorithm. We note that the Mailman algorithm is only 3-4 times faster than the basic EM algorithm instead of the log factor predicted by theory. We suspect that a reason for this gap is that the Mailman algorithm, as implemented, has not been optimized for specific computing architectures unlike standard matrix algorithms.

References

1. Goddard P Kichaev G Gusev A Pasaniuc B Mancuso N, Shi H. Integrating gene expression with summary association statistics to identify genes associated with 30 complex traits. *Am J Hum Genet*, 100(3):473–487, 2017.

2. Mallen C Zhang W Doherty M Kuo CF, Grainge MJ. Rising burden of gout in the uk but continuing suboptimal management: a nationwide population study. *Annals of the Rheumatic Diseases*, 74:661–667, 2015.

3. Richard A. Sturm, David L. Duffy, Zhen Zhen Zhao, Fabio P.N. Leite, Mitchell S. Stark, Nicholas K. Hayward, Nicholas G. Martin, and Grant W. Montgomery. A single snp in an evolutionary conserved region within intron 86 of the herc2 gene determines human blue-brown eye color. *AJHG*, 82(2):424–31, 2008.

4. Iain Mathieson, Iosif Lazaridis, Nadin Rohland, Swapan Mallick, Nick Patterson, Songül Alpaslan Roodenberg, Eadaoin Harney, Kristin Stewardson, Daniel Fernandes, Mario Novak, et al. Genome-wide patterns of selection in 230 ancient eurasians. *Nature*, 528(7583):499–503, 2015.

5. Janssens AC Rivadeneira F Lao O van Duijn K Vermeulen M Arp P Jhamai MM van Ijcken WF den Dunnen JT Heath S Zelenika D Despriet DD Klaver CC Vingerling JR de Jong PT Hofman A Aulchenko YS Uitterlinden AG Oostra BA van Duijn CM Kayser M, Liu F. Three genome-wide association studies and a linkage analysis identify herc2 as a human iris color gene. *AJHG*, 82(2):411–23, 2008.

6. Lin Q. Chen D. et al. Reed, J.A. Ski pathways inducing progression of human melanoma. *Cancer and Metastasis Reviews*, 24(2):265–272, 2005.

7. W Chen, SS Lam, H Srinath, CA Schiffer, WE Royer, and K Jr Lin. Competition between ski and creb-binding protein for binding to smad proteins in transforming growth factor-β signaling", journal="journal of biological chemistry. 282(15):11365–11376, 2007.

8. Zhang J Zhang J Li X Xie M, Wu X. Ski regulates smads and taz signaling to suppress lung cancer progression. *Journal of Biological Chemistry*, 56(10):2178–2189, 2017.

9. P De Camilli, A Thomas, R Cofiell, F Folli, B Lichte, G Piccolo, H M Meinck, M Austoni, G Fassetta, and G Bottazzo. The synaptic vesicle-associated protein amphiphysin is the 128-kd autoantigen of stiff-man syndrome with breast cancer. *Journal of Experimental Medicine*, 178(6):2219–2223, 1993.

10. Kevin J Galinsky, Gaurav Bhatia, Po-Ru Loh, Stoyan Georgiev, Sayan Mukherjee, Nick J Patterson, and Alkes L Price. Fast principal-component analysis reveals convergent evolution of adh1b in europe and east asia. *The American Journal of Human Genetics*, 98(3):456–472, 2016.

11. Nicolas Duforet-Frebourg, Keurcien Luu, Guillaume Laval, Eric Bazin, and Michael G.B. Blum. Detecting genomic signatures of natural selection with principal component analysis: Application to the 1000 genomes data. *Mol Biol Evol*, 33(4):1082–1093, 2016.

12. Michael G. B. Blum Keurcien Luu, Eric Bazin. pcadapt: an r package to perform genome scans for selection based on principal component analysis. *Molecular Ecology Resources*, 17(1):67–77, 2017.

13. Albers and McVean. Dating genomic variants and shared ancestry in population-scale sequencing data. *bioRxiv*, 2019.

14. The 1000 Genomes Project Consortium. An integrated map of genetic variation from 1,092 human genomes. *Nature*, 491(7422):56–65, Nov 2012.

15. Yair Field, Evan A Boyle, Natalie Telis, Ziyue Gao, Kyle J. Gaulton, David Golan, Loic Yengo, Ghislain Rocheleau, Philippe Froguel, Mark I. McCarthy, and Jonathan K. Pritchard. Detection of human adaptation during the past 2000 years. *Science*, 354(6313):760–764, 2016.

16. Roderick JA Little and Donald B Rubin. *Statistical analysis with missing data*. John Wiley & Sons, 2014.

17. Edo Liberty and Steven W Zucker. The mailman algorithm: A note on matrix–vector multiplication. *Information Processing Letters*, 109(3):179–182, 2009.

18. CF Jeff Wu. On the convergence properties of the em algorithm. *The Annals of statistics*, pages 95–103, 1983.

19. Forrest W Young, Yoshio Takane, and Jan de Leeuw. The principal components of mixed measurement level multivariate data: An alternating least squares method with optimal scaling features. *Psychometrika*, 43(2):279–281, 1978.

20. Gaël Guennebaud, Benoît Jacob, et al. Eigen v3. http://eigen.tuxfamily.org, 2010.
