# Supplementary material for: Scalable probabilistic PCA for large-scale genetic variation data
Source: PLoS Genet. 2020 May 29;16(5):e1008773. doi: 10.1371/journal.pgen.1008773 (PMC7286535; doi:10.1371/journal.pgen.1008773)
Supplement: S1 Fig — Comparison of methods when calculating differing numbers of principal components. We computed principal components ranging from 1-40 on a dataset containing 20 populations separated at Fst = 0.01, 10, 000 individuals, and 50, 000 SNPs. All methods were run with default settings. (PDF) [file pgen.1008773.s002.pdf]

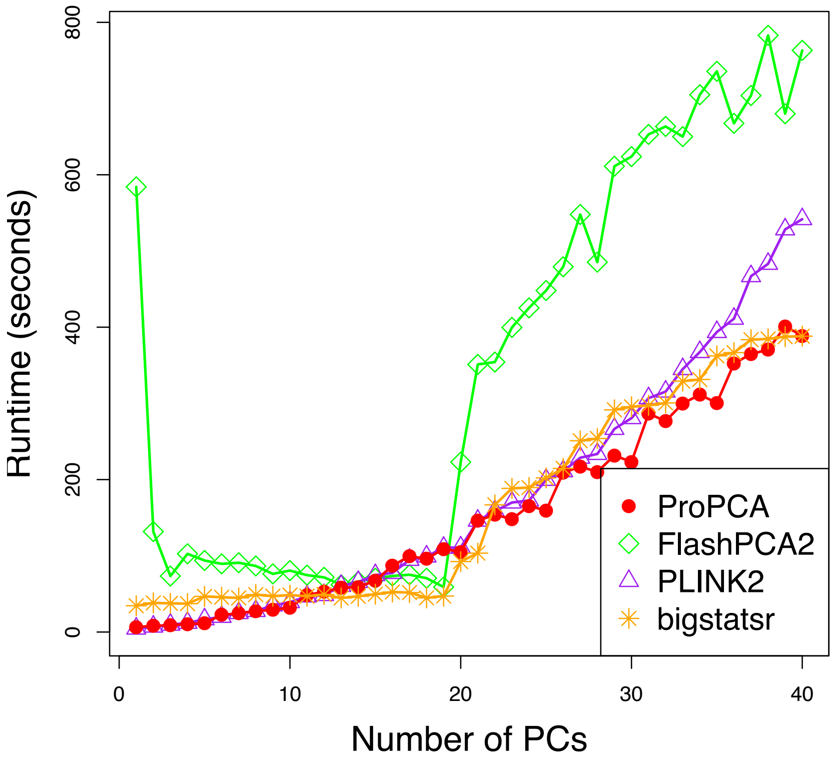

Figure S1: **ProPCA is efficient at computing large numbers of PCs.** Comparison of methods when calculating differing numbers of principal components. We computed principal components ranging from 1-40 on a dataset containing 20 populations separated at $F_{st} = 0.01$, $10,000$ individuals, and $50,000$ SNPs. All methods were run with default settings.
