# Supplementary material for: Scalable probabilistic PCA for large-scale genetic variation data
Source: PLoS Genet. 2020 May 29;16(5):e1008773. doi: 10.1371/journal.pgen.1008773 (PMC7286535; doi:10.1371/journal.pgen.1008773)
Supplement: S2 Fig — Comparison of average per-iteration runtimes over simulated genotype data containing 100, 000 SNPs, six subpopulations, Fst = 0.10 and individuals varying from 10, 000 to 1, 000, 00. We were unable to leverage the source code for FlashPCA2 for this comparison. (PDF) [file pgen.1008773.s003.pdf]

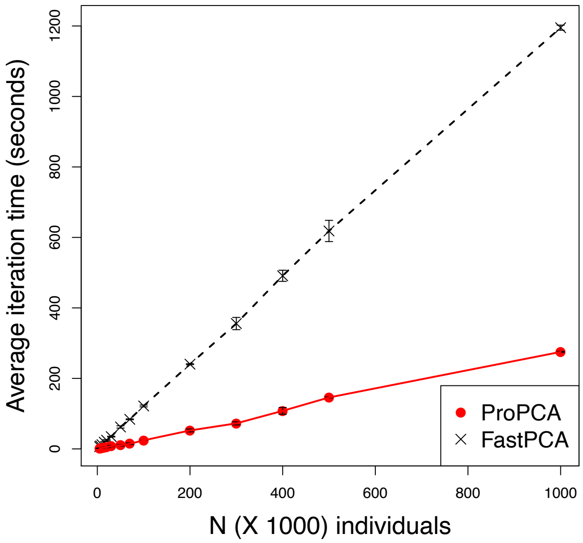

Figure S2: **ProPCA has faster per-iteration runtimes versus FastPCA**: Comparison of average per-iteration runtimes over simulated genotype data containing 100,000 SNPs, six subpopulations, $F_{st} = 0.10$ and individuals varying from 10,000 to 1,000,00. We were unable to leverage the source code for FlashPCA2 for this comparison.
