# Supplementary material for: Scalable probabilistic PCA for large-scale genetic variation data
Source: PLoS Genet. 2020 May 29;16(5):e1008773. doi: 10.1371/journal.pgen.1008773 (PMC7286535; doi:10.1371/journal.pgen.1008773)
Supplement: S3 Fig — We compute the total time taken to estimate the top five principal components as a function of a measure of accuracy (MEV) for ProPCA compared to FastPCA and FlashPCA2. We performed these comparisons on simulated genotype data containing 50, 000 SNPs, 10, 000 individuals, six subpopulations, and Fst ∈ {0.001, 0.005, 0.01}. (PDF) [file pgen.1008773.s004.pdf]

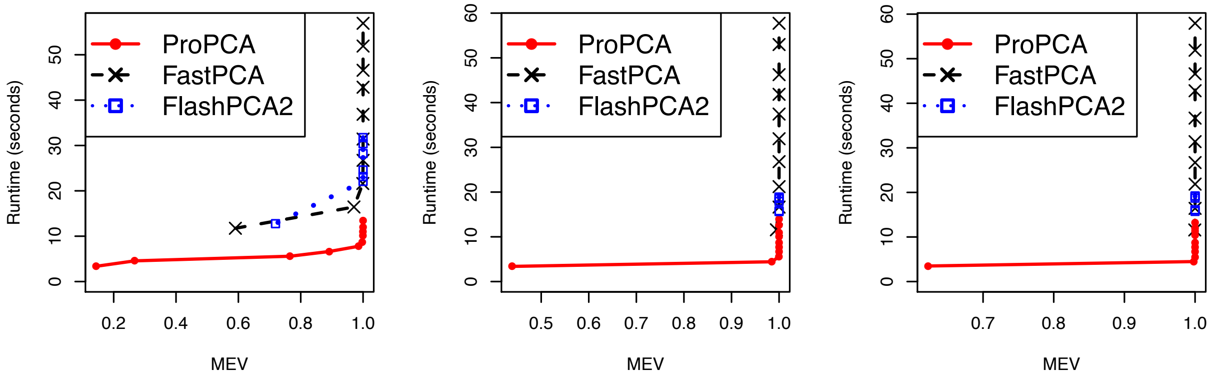

Figure S3: **ProPCA is computationally efficient relative to other methods**: We compute the total time taken to estimate the top five principal components as a function of a measure of accuracy (MEV) for ProPCA compared to FastPCA and FlashPCA2. We performed these comparisons on simulated genotype data containing 50,000 SNPs, 10,000 individuals, six subpopulations, and $F_{st} \in \{0.001, 0.005, 0.01\}$.
