# Supplementary material for: Scalable probabilistic PCA for large-scale genetic variation data
Source: PLoS Genet. 2020 May 29;16(5):e1008773. doi: 10.1371/journal.pgen.1008773 (PMC7286535; doi:10.1371/journal.pgen.1008773)
Supplement: S4 Fig — We display the memory usage in gigabytes of each method when computing the top 5 principal components. S4 Figa show the average memory usage from each method over 10 runs on a dataset containing six populations separated at FST = 0.01, 100, 000 SNPs, and individuals varying from 100,000 to 1,000,000. Figure S4 Figb shows a similar result, but with 100, 000 individuals and SNPs varying from 100,000 to 1,000,000 over a single run. All methods were run using default settings. We were unable to run bigstatsr for the SNPs experiment due to a bug that causes the method to crash in the presence of monomorphic SNPs. (PDF) [file pgen.1008773.s005.pdf]

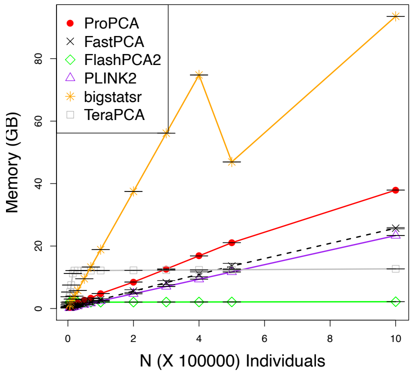

(a)

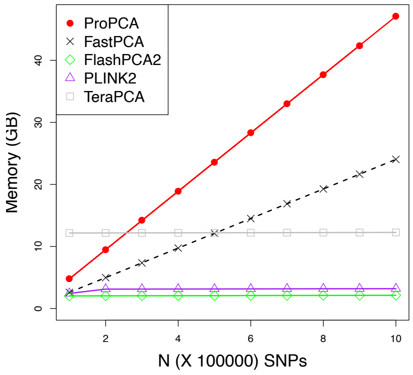

(b)

Figure S4: **ProPCA memory usage scales linearly.** We display the memory usage in gigabytes of each method when computing the top 5 principal components. Figure S4a show the average memory usage from each method over 10 runs on a dataset containing six populations separated at $F_{ST} = 0.01$, 100,000 SNPs, and individuals varying from 100,000 to 1,000,000. Figure S4b shows a similar result, but with 100,000 individuals and SNPs varying from 100,000 to 1,000,000 over a single run. All methods were run using default settings. We were unable to run bigstatsr for the SNPs experiment due to inability to run with monomorphic SNPs.
