# Supplementary material for: Scalable probabilistic PCA for large-scale genetic variation data
Source: PLoS Genet. 2020 May 29;16(5):e1008773. doi: 10.1371/journal.pgen.1008773 (PMC7286535; doi:10.1371/journal.pgen.1008773)
Supplement: S5 Fig — We applied our method to genotype data from Phase 1 of the 1000 Genomes project. On a dataset of 1, 092 individuals and 442, 350 SNPs, ProPCA computes the top five PCs in about 17 seconds on a single core. The top two PCs computed by ProPCA and by running SVD on this data set are qualitatively indistinguishable. EM refers to ProPCA. (PDF) [file pgen.1008773.s006.pdf]

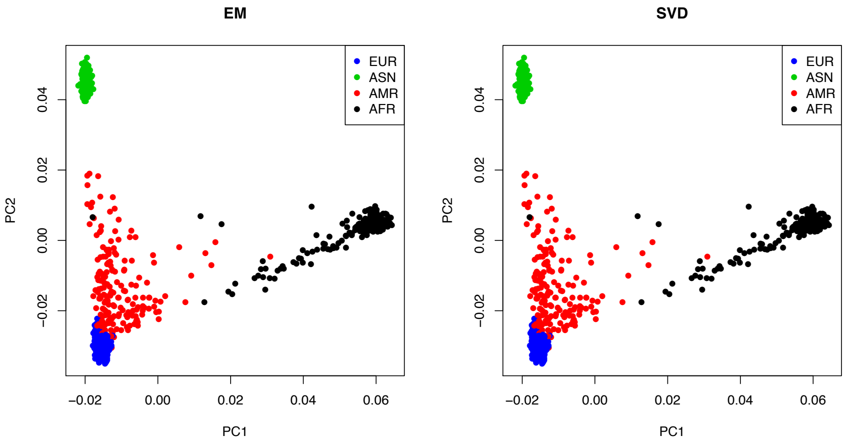

Figure S5: **ProPCA estimates principal components that are qualitatively indistinguishable from a full SVD on 1000 Genomes Phase 1 data.** We applied our method to genotype data from Phase 1 of the 1000 Genomes project. On a dataset of 1,092 individuals and 442,350 SNPs, ProPCA computes the top five PCs in about 17 seconds on a single core. The top two PCs computed by ProPCA and by running SVD on this data set are qualitatively indistinguishable. EM refers to ProPCA.
