# Supplementary material for: Scalable probabilistic PCA for large-scale genetic variation data
Source: PLoS Genet. 2020 May 29;16(5):e1008773. doi: 10.1371/journal.pgen.1008773 (PMC7286535; doi:10.1371/journal.pgen.1008773)
Supplement: S6 Fig — Plotting pairs of the first five principal components reveals structure amongst the unrelated White British. This structure diminishes as we increase the number principal components used. (PDF) [file pgen.1008773.s007.pdf]

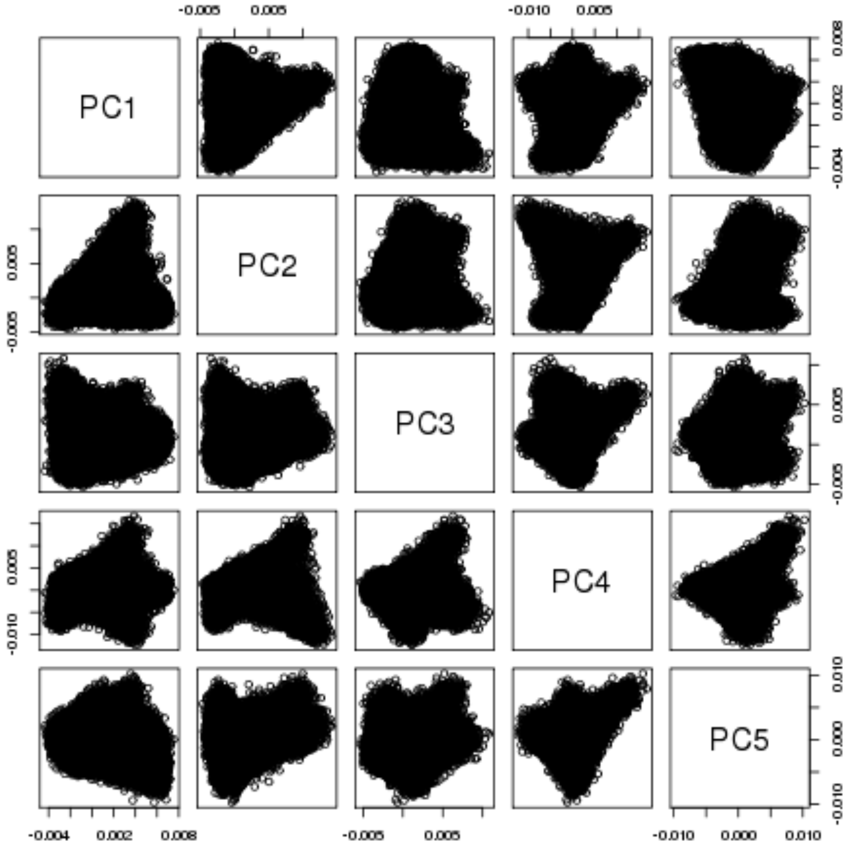

Figure S6: **Scatterplot pairs between the projections of the first five principal components of the unrelated White British**: Plotting pairs of the first five principal components reveals structure amongst the unrelated White British. This structure diminishes as we increase the number principal components used.
