# Supplementary material for: Scalable probabilistic PCA for large-scale genetic variation data
Source: PLoS Genet. 2020 May 29;16(5):e1008773. doi: 10.1371/journal.pgen.1008773 (PMC7286535; doi:10.1371/journal.pgen.1008773)
Supplement: S7 Fig — Using birth location data available in the UK Biobank, we placed a scatter plot colored by principal component score to reveal geographic variation captured by the principal components. (PDF) [file pgen.1008773.s008.pdf]

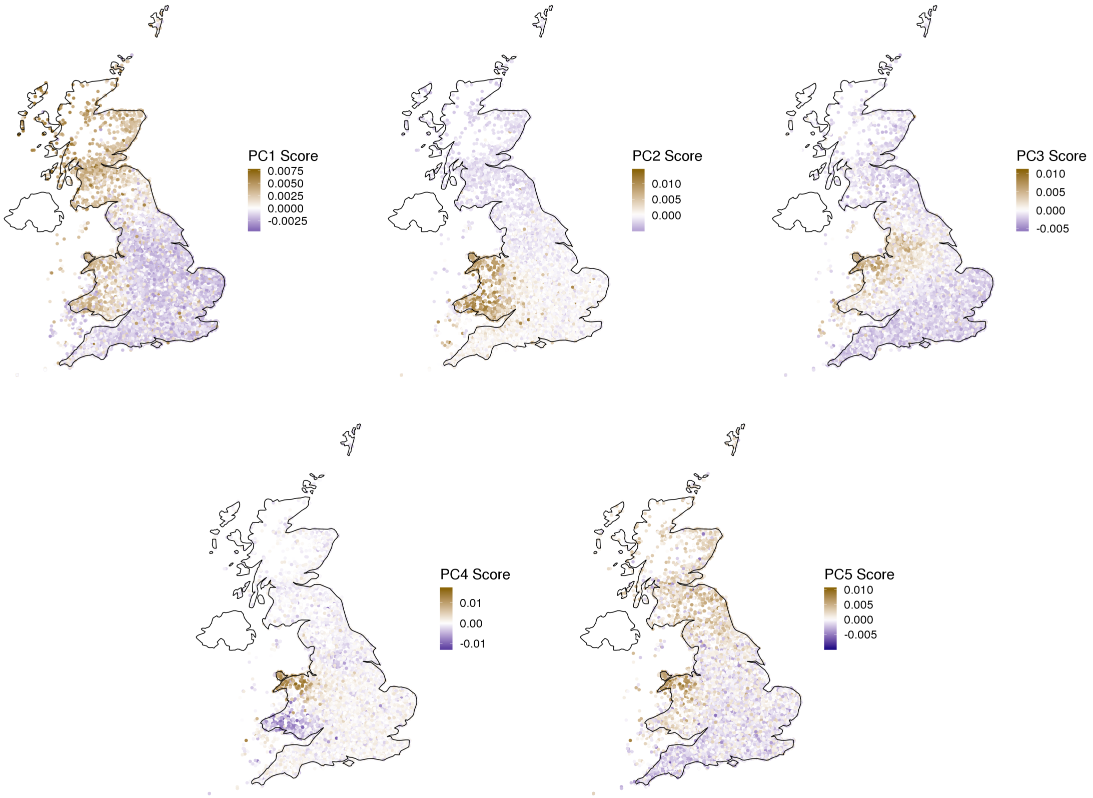

Figure S7: **Principal component scores of the unrelated White British overlaid on a map of the UK**: Using birth location data available in the UK Biobank, we placed a scatter plot colored by principal component score to reveal geographic variation captured by the principal components.
