# Supplementary material for: Scalable probabilistic PCA for large-scale genetic variation data
Source: PLoS Genet. 2020 May 29;16(5):e1008773. doi: 10.1371/journal.pgen.1008773 (PMC7286535; doi:10.1371/journal.pgen.1008773)
Supplement: S8 Fig — We plot the theoretical quantiles of the χ12 distribution against each of the empirical quantiles observed from the first five principal components. All five principal components follow the theoretical distribution well until the upper tail. We additionally show the calibration of the combined statistic against the theoretical quantiles of the χ52 distribution. (PDF) [file pgen.1008773.s009.pdf]

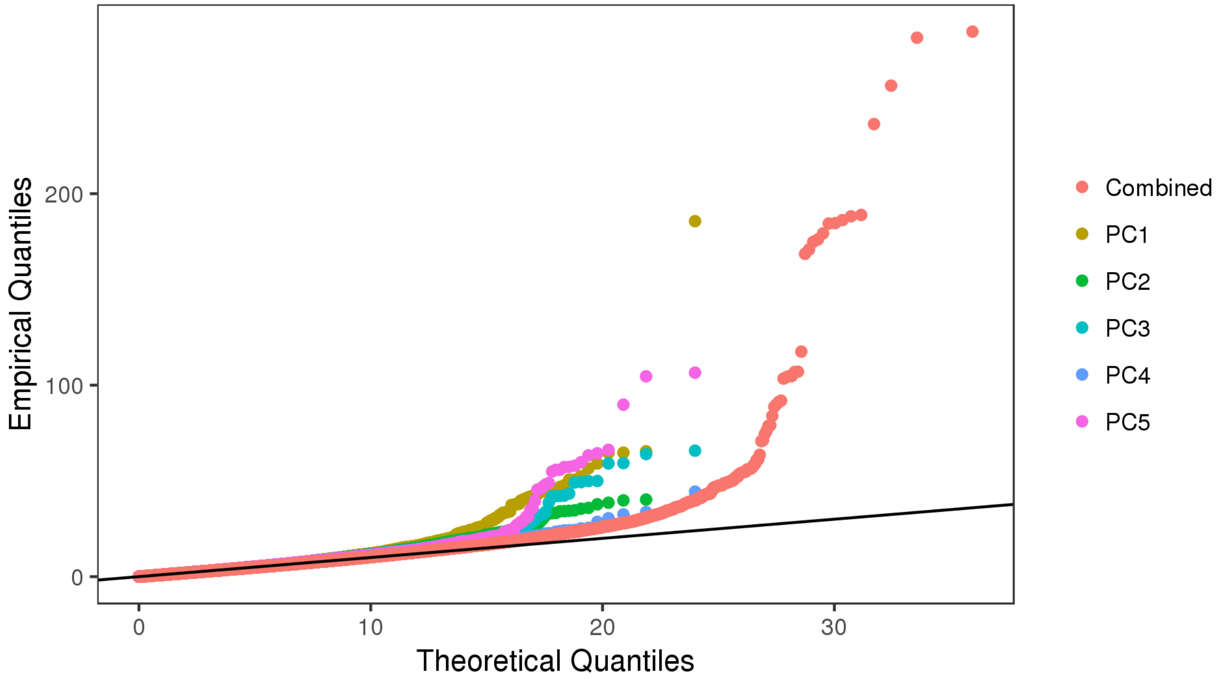

Figure S8: **The selection statistic is calibrated in the unrelated White British**: We plot the theoretical quantiles of the χ_1^2 distribution against each of the empirical quantiles observed from the first five principal components. All five principal components follow the theoretical distribution well until the upper tail. We additionally show the calibration of the combined statistic against the theoretical quantiles of the χ_5^2 distribution.
