# Supplementary material for: Scalable probabilistic PCA for large-scale genetic variation data
Source: PLoS Genet. 2020 May 29;16(5):e1008773. doi: 10.1371/journal.pgen.1008773 (PMC7286535; doi:10.1371/journal.pgen.1008773)
Supplement: S9 Fig — Using allelic age estimates from the Human Genome Dating Atlas of Variant Age, we compared the estimated allelic ages of the significant signals of selection in Field et al. 2016 (SDS score > 4) and signals found by our own selection statistic. The x-axis denotes different clock models used to estimate allelic ages while allelic age estimates are denoted in generations on the y-axis. The joint clock model estimates allelic age using information from both the recombination and mutational clock models. (PDF) [file pgen.1008773.s010.pdf]

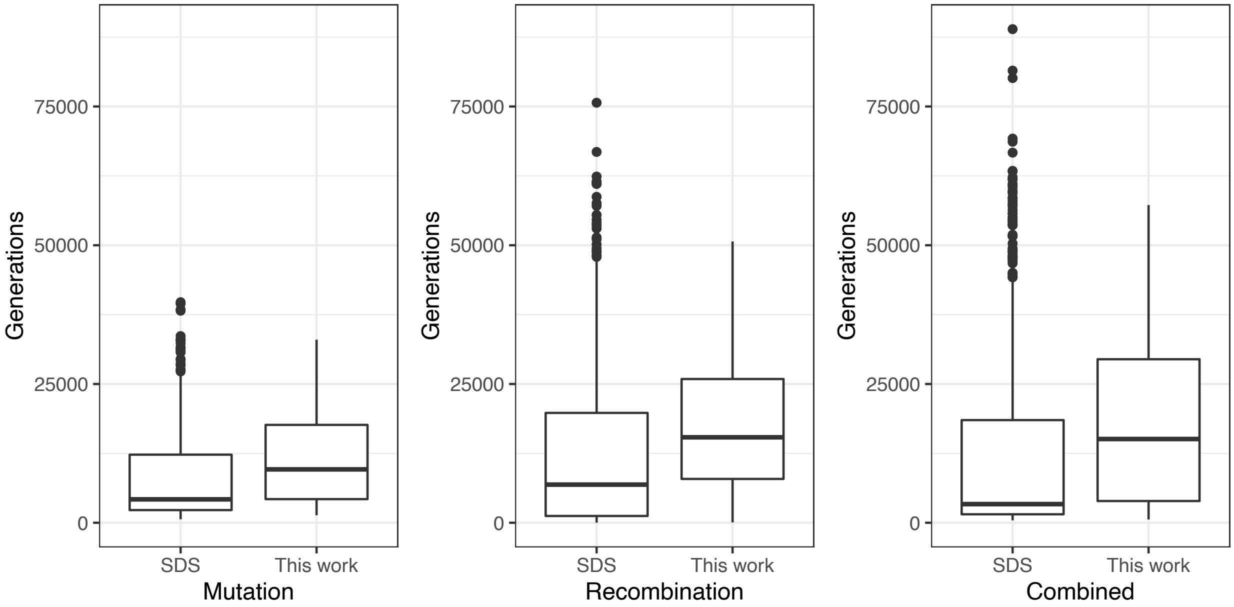

Figure S9: **Boxplot of estimated allelic ages of putative signals of selection**: Using allelic age estimates from the Human Genome Dating Atlas of Variant Age, we compared the estimated allelic ages of the significant signals of selection in Field et al. 2016 (SDS score > 4) and signals found by our own selection statistic. The x-axis denotes different clock models used to estimate allelic ages while allelic age estimates are denoted in generations on the y-axis. The joint clock model estimates allelic age using information from both the recombination and mutational clock models.
