# Supplementary material for: Scalable probabilistic PCA for large-scale genetic variation data
Source: PLoS Genet. 2020 May 29;16(5):e1008773. doi: 10.1371/journal.pgen.1008773 (PMC7286535; doi:10.1371/journal.pgen.1008773)
Supplement: S10 Fig — To further illustrate the importance of large sample sizes for biological discovery, we analyzed how many selection signals we could discover as a function of sample size. We randomly subsampled 10,000, 50,000, 100,000, and 200,000 individuals from the White British populations and performed our selection scan. The x-axis denotes sample size in thousands and the y-axis denotes the proportions of total hits discovered. (PDF) [file pgen.1008773.s011.pdf]

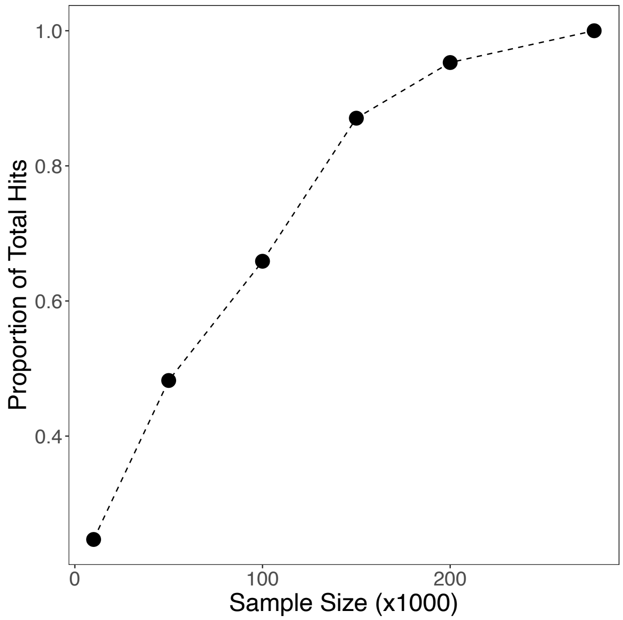

Figure S10: **Proportion of total number of selection signal hits as a function of sample size**: To further illustrate the importance of large sample sizes for biological discovery, we analyzed how many selection signals we could discover as a function of sample size. We randomly subsampled 10,000, 50,000, 100,000, and 200,000 individuals from the White British populations and performed our selection scan. The x-axis denotes sample size in thousands and the y-axis denotes the proportions of total hits discovered.
