# Supplementary material for: Scalable probabilistic PCA for large-scale genetic variation data
Source: PLoS Genet. 2020 May 29;16(5):e1008773. doi: 10.1371/journal.pgen.1008773 (PMC7286535; doi:10.1371/journal.pgen.1008773)
Supplement: S11 Fig — We compared the MEV from eigenvectors calculated from both modes of ProPCA with ground truth eigenvectors from performing a full SVD. We evaluated performance at 5% and 20% random missing values at 5 (S11a Fig) and 10 principal components (S11b Fig). We additionally compared ProPCA to mean imputation followed by a full SVD (S11c Fig). The data consists of simulated genotype data of 50, 000 SNPs from 10, 000 individuals from 5 populations for 5 PCs and 10 populations for 10 PCs separated by a range of Fst values. This process was repeated ten times to measure variability. Error bars denoting one standard deviation are shown for each point. (PDF) [file pgen.1008773.s012.pdf]

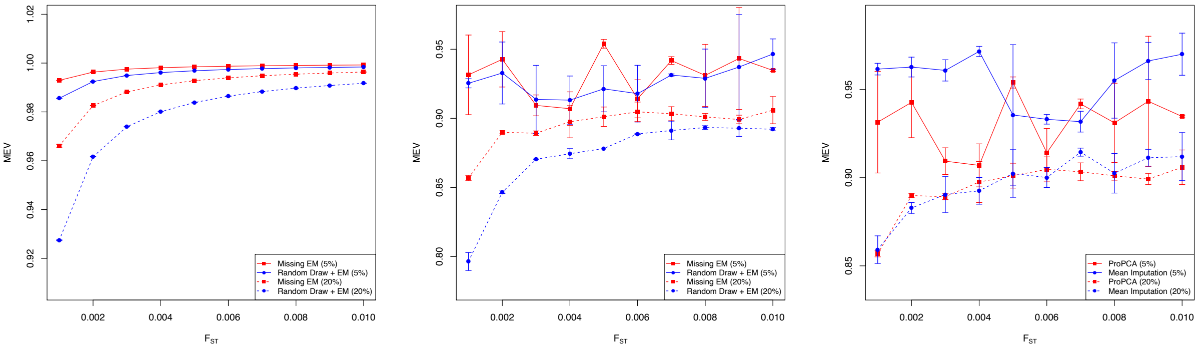

(a) (b) (c)

Figure S11: **ProPCA infers more accurate principal components (PCs) in the presence of missing data compared to imputed genotypes**: We compared the MEV from eigenvectors calculated from both modes of ProPCA with ground truth eigenvectors from performing a full SVD. We evaluated performance at 5% and 20% random missing values at 5 (S11a) and 10 principal components (S11b). We additionally compared ProPCA to mean imputation followed by a full SVD (S11c). The data consists of simulated genotype data of $50,000$ SNPs from $10,000$ individuals from 5 populations for 5 PCs and 10 populations for 10 PCs separated by a range of F_{st} values. This process was repeated ten times to measure variability. Error bars denoting one standard deviation are shown for each point.
