# Supplementary material for: Scalable probabilistic PCA for large-scale genetic variation data
Source: PLoS Genet. 2020 May 29;16(5):e1008773. doi: 10.1371/journal.pgen.1008773 (PMC7286535; doi:10.1371/journal.pgen.1008773)
Supplement: S12 Fig — We compare the time taken to compute the top five principal components by the EM algorithm underlying ProPCA when used in conjunction with the Mailman algorithm and without. We performed these comparisons on simulated genotype data containing 100, 000 SNPs, six subpopulations, Fst = 0.10 and individuals varying from 10, 000 to 1, 000, 000. S12 Figa compares the runtime of the EM algorithm with the Mailman matrix-vector multiplication to an EM algorithm where the genotypes are represented as a matrix of doubles (EM1). With this representation, the EM algorithm could only be applied to sample sizes of at most 70, 000 individuals due to memory constraints. S12 Figb compares the runtime of the EM algorithm with the Mailman matrix-vector multiplication to an EM algorithm where genotypes are represented in a compact representation (EM2). (PDF) [file pgen.1008773.s013.pdf]

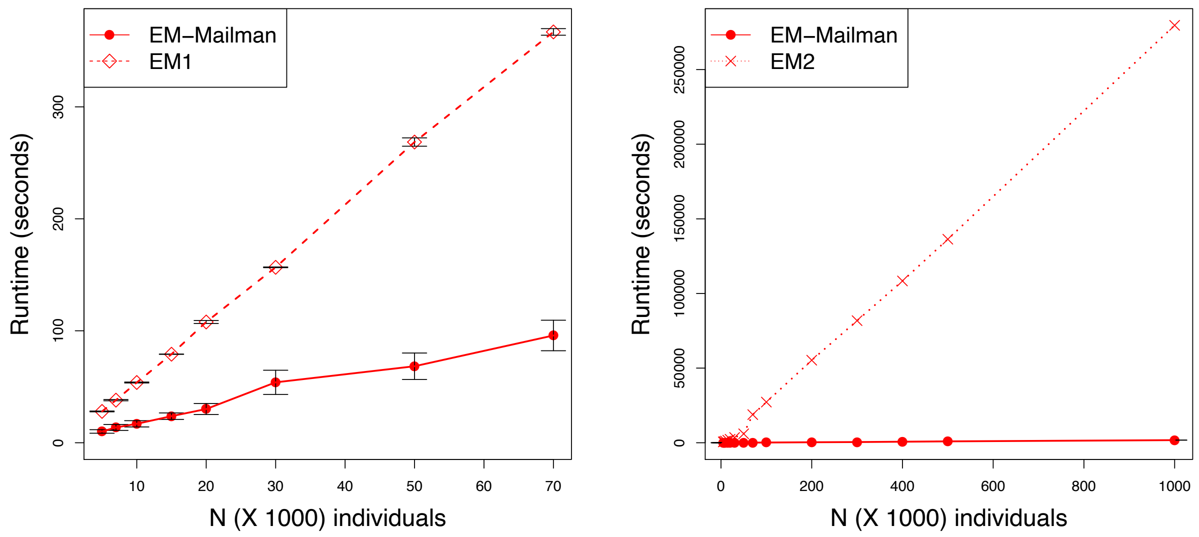

(a) (b)

Figure S12: **The Mailman matrix-vector multiplication contributes to the scalability of ProPCA**: We compare the time taken to compute the top five principal components by the EM algorithm underlying ProPCA when used in conjunction with the Mailman algorithm and without. We performed these comparisons on simulated genotype data containing $100,000$ SNPs, six subpopulations, $F_{st} = 0.10$ and individuals varying from $10,000$ to $1,000,000$. Figures S12a compares the runtime of the EM algorithm with the Mailman matrix-vector multiplication to an EM algorithm where the genotypes are represented as a matrix of doubles (EM1). With this representation, the EM algorithm could only be applied to sample sizes of at most $70,000$ individuals due to memory constraints. Figure S12b compares the runtime of the EM algorithm with the Mailman matrix-vector multiplication to an EM algorithm where genotypes are represented in a compact representation (EM2).
