# Supplementary material for: Scalable probabilistic PCA for large-scale genetic variation data
Source: PLoS Genet. 2020 May 29;16(5):e1008773. doi: 10.1371/journal.pgen.1008773 (PMC7286535; doi:10.1371/journal.pgen.1008773)
Supplement: S1 Table — We compared the accuracy of the ProPCA algorithm, bigsnpr, FlashPCA2, PLINK2, TeraPCA, and FastPCA when applied to 1092 individuals in the 1000 Genomes Phase 1 project. We report MEV averaged over ten trials. FastPCA gave us a segmentation fault for estimation of ≥ 35 PCs. We ran all methods using default settings. (PDF) [file pgen.1008773.s014.pdf]

	**PC5**	**PC10**	**PC15**	**PC20**	**PC25**	**PC30**	**PC35**	**PC40**
**bigsnpr**	1.0000	1.0000	1.0000	1.0000	1.0000	1.0000	1.0000	1.0000
**FlashPCA2**	1.0000	1.0000	1.0000	1.0000	1.0000	1.0000	1.0000	1.0000
**PLINK2**	0.9816	1.0000	0.9990	0.9999	0.9981	0.9998	0.9999	1.0000
**ProPCA**	0.9825	0.9994	0.9932	0.9975	0.9991	0.9999	1.0000	1.0000
**TeraPCA**	0.9996	0.9943	0.6714	0.5000	0.9247	0.9505	0.9429	0.9548
**FastPCA**	1.0000	1.0000	1.0000	1.0000	1.0000	1.0000	NA	NA

Table S1: **Comparison of accuracy of methods to estimate principal components on the genotype data from the 1000 Genomes Phase 1 project**. We compared the accuracy of the ProPCA algorithm, bigsnpr, FlashPCA2, PLINK2, TeraPCA, and FastPCA when applied to 1092 individuals in the 1000 Genomes Phase 1 project. We report MEV averaged over ten trials. FastPCA gave us a segmentation fault for estimation of ≥ 35 PCs. We ran all methods using default settings.
