# Supplementary material for: Scalable probabilistic PCA for large-scale genetic variation data
Source: PLoS Genet. 2020 May 29;16(5):e1008773. doi: 10.1371/journal.pgen.1008773 (PMC7286535; doi:10.1371/journal.pgen.1008773)
Supplement: S2 Table — Pearson correlation between the principal components and birth location coordinates reveals that the principal components unveil geographic variation. P-values from Pearson correlation t-test is shown in parentheses on the right. (PDF) [file pgen.1008773.s015.pdf]

	PC1	PC2	PC3	PC4	PC5
**Longitude**	-0.39 (0)	-0.06 (6.29e-188)	-0.18 (0)	0.03 (4.88e-42)	-0.11 (0)
**Latitude**	0.38 (0)	-0.41 (0)	0.16 (0)	-0.05 (1.06e-141)	0.32 (0)

Table S2: **Pearson correlation between principal components and birth location coordinates in the unrelated White British.** Pearson correlation between the principal components and birth location coordinates reveals that the principal components unveil geographic variation. P-values from Pearson correlation t-test is shown in parentheses on the right.
