# Supplementary material for: Scalable probabilistic PCA for large-scale genetic variation data
Source: PLoS Genet. 2020 May 29;16(5):e1008773. doi: 10.1371/journal.pgen.1008773 (PMC7286535; doi:10.1371/journal.pgen.1008773)
Supplement: S3 Table — We calculated the λGC values for each principal component and the combined statistic to check for inflation. In the unrelated White British set, the calculated values show that our selection statistics are not substantially inflated (top row). Furthermore, we show that the previously related statistic proposed by Galinsky et al. 2016 does not calibrate as well as our statistics based on λGC values (bottom row). (PDF) [file pgen.1008773.s016.pdf]

λ_{GC}	**PC1**	**PC2**	**PC3**	**PC4**	**PC5**	**Combined**
**Statistic**	0.961	0.970	0.979	0.955	0.958	0.962
**Galinsky**	1.017	0.904	0.900	0.791	0.794	0.877

Table S3: **Selection statistics are not substantially inflated.** We calculated the λ_{GC} values for each principal component and the combined statistic to check for inflation. In the unrelated White British set, the calculated values show that our selection statistics are not substantially inflated (top row). Furthermore, we show that the previously related statistic proposed by Galinsky et al. 2016 does not calibrate as well as our statistics based on λ_{GC} values (bottom row).
