# Supplementary material for: Scalable probabilistic PCA for large-scale genetic variation data
Source: PLoS Genet. 2020 May 29;16(5):e1008773. doi: 10.1371/journal.pgen.1008773 (PMC7286535; doi:10.1371/journal.pgen.1008773)
Supplement: S5 Table — We obtained 59 selection hits across the first five principal components of the unrelated White British subset of the UK Biobank. We clustered these hits into 12 unique loci by aggregating all significant hits into 1 Mb windows centered around the most significant hits. Other genes with significant hits that are within the 1 Mb window are listed in the last column. (PDF) [file pgen.1008773.s018.pdf]

CHR	POS	rsid	Gene	PC1	Other Gene Hits in Window
2	135837906	rs7570971	RAB3GAP1	2.64E-09	RAB3GAP1,R3HDM1,LCT
4	38799710	rs4833095	TLR1	1.80E-09	TLR10,TLR1,TLR6,FAM114A1
6	421281	rs62389423		8.80E-36	IRF4,EXOC2
6	32139813	rs9267817		0.019121	HLA
19	49206417	rs492602	FUT2	0.002174	FUT2
**CHR**	**POS**	**rsid**	**Gene**	**PC2**	**Other Gene Hits in Window**
1	2240074	rs79907870	SKI	0.007996	SKI
1	116977051	rs6670894		0.015407	
4	38799710	rs4833095	TLR1	0.000341	TLR10,TLR1,FAM114A1
5	164861910	rs77635680		0.000687	
9	13954710	rs12380860		0.026696	
15	28365618	rs12913832	HERC2	0.001613	HERC2
16	53720436	rs61747071	RPGRIP1L	0.006486	RPGRIP1L
**CHR**	**POS**	**rsid**	**Gene**	**PC3**	**Other Gene Hits in Window**
2	136407479	rs1446585	R3HDM1	0.001585	R3HDM1,LCT
4	38799710	rs4833095	TLR1	1.56E-09	TLR10,TLR1,TLR6,FAM114A1
**CHR**	**POS**	**rsid**	**Gene**	**PC4**	**Other Gene Hits in Window**
4	89323743	rs112873858	HERC6	8.29E-05	HERC6
5	164847509	rs79194719		0.019347	
**CHR**	**POS**	**rsid**	**Gene**	**PC5**	**Other Gene Hits in Window**
4	38798935	rs5743614	TLR1	1.75E-18	TLR10,TLR1,TLR6,FAM114A1
6	421281	rs62389423		0.006962	

Table S5: **Principal component selection scan reveals 12 unique loci under selection across the top five principal components.** We obtained 59 selection hits across the first five principal components of the unrelated White British subset of the UK Biobank. We clustered these hits into 12 unique loci by aggregating all significant hits into 1 Mb windows centered around the most significant hits. Other genes with significant hits that are within the 1 Mb window are listed in the last column.
