# Supplementary material for: Scalable probabilistic PCA for large-scale genetic variation data
Source: PLoS Genet. 2020 May 29;16(5):e1008773. doi: 10.1371/journal.pgen.1008773 (PMC7286535; doi:10.1371/journal.pgen.1008773)
Supplement: S6 Table — We performed a two-tailed proportion test for our novel loci between the allele frequency in each individual region from the NUTS3 classification of the United Kingdom against the frequency from every other region. We corrected the p-values using the Bonferroni correction (11 loci × 163 regions). The corrected p-values for regions passing the significance threshold are shown in the table. (PDF) [file pgen.1008773.s019.pdf]

SNP	Genes in Window	P	Phenotype
rs12913832	HERC2	0	pigment_HAIR_blackmale
		0	pigment_HAIR_blonde
		0	pigment_HAIR_darkbrown
		0	pigment_HAIR
		9.70E-103	pigment_HAIR_red
		0	pigment_SKIN
		1.50E-138	pigment_SUNBURN
		0	pigment_TANNING
rs492602	FUT2	2.50E-09	blood_HIGH_LIGHT_SCATTER_RETICULOCYTE_COUNT
		1.80E-53	blood_MEAN_PLATELET_VOL
		5.20E-11	blood_MEAN_SPHERED_CELL_VOL
		9.70E-18	blood_PLATELET_COUNT
		1.10E-08	body_HEIGHTz
		7.50E-13	bp_DIASTOLICadjMEDz
		1.20E-12	bp_SYSTOLICadjMEDz
		9.40E-19	disease_CARDIOVASCULAR
		2.60E-21	disease_HI_CHOL_SELF_REP
		1.60E-09	disease_HYPERTENSION_DIAGNOSED
		8.80E-12	lung_FEV1FVCzSMOKE
rs62389423	IRF4,EXOC2	6.80E-29	blood_EOSINOPHIL_COUNT
		4.70E-19	blood_LYMPHOCYTE_COUNT
		2.20E-16	blood_WHITE_COUNT
		2.40E-68	body_BALDING1
		2.00E-66	body_BALDING4
		3.20E-31	cancer_ALL
		0	pigment_HAIR_blackmale
		0	pigment_HAIR_blonde
		0	pigment_HAIR_darkbrown
		0	pigment_HAIR
		1.90E-33	pigment_HAIR_red
		0	pigment_SKIN
		0	pigment_SUNBURN
		0	pigment_TANNING
rs7570971	RAB3GAP1,R3HDM1,LCT	1.90E-08	blood_EOSINOPHIL_COUNT
		1.70E-09	blood_RED_COUNT
		2.60E-15	lung_FVCzSMOKE
rs9267817	HLA	2.50E-10	blood_MEAN_PLATELET_VOL
		6.30E-13	blood_MONOCYTE_COUNT
		3.70E-16	blood_RBC_DISTRIB_WIDTH
		1.00E-13	body_HEIGHTz
		7.00E-10	bp_SYSTOLICadjMEDz
		2.40E-13	impedance_BASAL_METABOLIC_RATEz
		6.10E-27	lung_FEV1FVCzSMOKE

Table S6: **Selection hits are associated with phenotypes in the UK Biobank.** We ran genome-wide association tests for 64 phenotypes in the full release of the UK Biobank for each of our loci. Phenotypes shown reached a p-value of genome-wide significance level (0.05×10^{-6}).
