# Supplementary material for: Scalable probabilistic PCA for large-scale genetic variation data
Source: PLoS Genet. 2020 May 29;16(5):e1008773. doi: 10.1371/journal.pgen.1008773 (PMC7286535; doi:10.1371/journal.pgen.1008773)
Supplement: S7 Table — We discover four additional novel loci using our combined selection statistic from the first five principal components. Loci not found in the individual PC selection statistics are denoted by an asterik in the rsid column. The chi-squared statistic (one degree of freedom) for each principal component is shown in the last five columns of the table. (PDF) [file pgen.1008773.s020.pdf]

CHR	POS	rsid	Gene	P	Other Gene Hits in Window	PC1	PC2	PC3	PC4	PC5
1	2240074	rs79907870	SKI	0.046830	SKI	1.27	35.48	4.63	2.15	1.38
2	136407479	rs1446585	R3HDM1	1.66E-14	RAB3GAP1,R3HDM1,UBXN4,LCT	56.55	2.40	38.63	2.12	5.02
3	54077256	rs9856661*		0.020002		0.94	9.18	12.43	0.72	23.45
4	89323743	rs112873858	HERC6	2.18E-05	HERC6	0.02	7.65	2.70	44.40	6.36
4	38799710	rs4833095	TLR1	6.22E-53	TLR10,TLR1,TLR6,FAM114A1	65.50	41.64	65.78	7.13	104.60
5	162948205	rs116352364*		0.000162		0.00	13.68	21.83	16.15	5.27
5	164861910	rs77635680		3.81E-11		2.81	40.26	5.00	32.52	8.16
6	32526736	rs111586361	HLA-DRB5	0.003826	HLA-DRB5	24.43	0.29	11.32	1.25	12.94
6	421281	rs62389423		7.11E-47	IRF4,EXOC2	185.64	13.97	12.09	8.97	35.75
7	38463542	rs118079376*	AMPH	0.000817	AMPH	1.08	0.01	6.36	18.80	27.26
9	120475302	rs4986790*	TLR4	2.35E-05	TLR4	20.37	25.98	1.68	0.67	12.29
15	28365618	rs12913832	HERC2	0.012037	HERC2	0.07	38.60	5.57	3.42	0.16
16	53720436	rs61747071	RPGRIP1L	0.012396	RPGRIP1L	0.76	35.88	4.67	6.39	0.05
19	49206417	rs492602	FUT2	0.000505	FUT2	38.02	12.08	0.33	3.03	1.08

Table S7: **Combined selection statistic across the top five principal components reveals four additional novel loci.** We discover four additional novel loci using our combined selection statistic from the first five principal components. Loci not found in the individual PC selection statistics are denoted by an asterik in the rsid column. The chi-squared statistic (one degree of freedom) for each principal component is shown in the last five columns of the table.
