# Supplementary material for: Scalable probabilistic PCA for large-scale genetic variation data
Source: PLoS Genet. 2020 May 29;16(5):e1008773. doi: 10.1371/journal.pgen.1008773 (PMC7286535; doi:10.1371/journal.pgen.1008773)
Supplement: S8 Table — We ran genome-wide association tests for 64 phenotypes in the full release of the UK Biobank for each of our loci. Phenotypes shown reached a p-value of genome-wide significance level (0.05 × 10^−6^). (PDF) [file pgen.1008773.s021.pdf]

SNP	Genes in Window	P	Phenotype
rs12913832	HERC2	0	pigment_HAIR_blackmale
		0	pigment_HAIR_blonde
		0	pigment_HAIR_darkbrown
		0	pigment_HAIR
		9.70E-103	pigment_HAIR_red
		0	pigment_SKIN
		1.50E-138	pigment_SUNBURN
		0	pigment_TANNING
rs492602	FUT2	2.50E-09	blood_HIGH_LIGHT_SCATTER_RETICULOCYTE_COUNT
		1.80E-53	blood_MEAN_PLATELET_VOL
		5.20E-11	blood_MEAN_SPHERED_CELL_VOL
		9.70E-18	blood_PLATELET_COUNT
		1.10E-08	body_HEIGHTz
		7.50E-13	bp_DIASTOLICadjMEDz
		1.20E-12	bp_SYSTOLICadjMEDz
		9.40E-19	disease_CARDIOVASCULAR
		2.60E-21	disease_HI_CHOL_SELF_REP
		1.60E-09	disease_HYPERTENSION_DIAGNOSED
		8.80E-12	lung_FEV1FVCzSMOKE
rs62389423	IRF4,EXOC2	6.80E-29	blood_EOSINOPHIL_COUNT
		4.70E-19	blood_LYMPHOCYTE_COUNT
		2.20E-16	blood_WHITE_COUNT
		2.40E-68	body_BALDING1
		2.00E-66	body_BALDING4
		3.20E-31	cancer_ALL
		0	pigment_HAIR_blackmale
		0	pigment_HAIR_blonde
		0	pigment_HAIR_darkbrown
		0	pigment_HAIR
		1.90E-33	pigment_HAIR_red
		0	pigment_SKIN
		0	pigment_SUNBURN
		0	pigment_TANNING
rs7570971	RAB3GAP1,R3HDM1,LCT	1.90E-08	blood_EOSINOPHIL_COUNT
		1.70E-09	blood_RED_COUNT
		2.60E-15	lung_FVCzSMOKE
rs9267817	HLA	2.50E-10	blood_MEAN_PLATELET_VOL
		6.30E-13	blood_MONOCYTE_COUNT
		3.70E-16	blood_RBC_DISTRIB_WIDTH
		1.00E-13	body_HEIGHTz
		7.00E-10	bp_SYSTOLICadjMEDz
		2.40E-13	impedance_BASAL_METABOLIC_RATEz
		6.10E-27	lung_FEV1FVCzSMOKE

Table S8: **Selection hits are associated with phenotypes in the UK Biobank.** We ran genome-wide association tests for 64 phenotypes in the full release of the UK Biobank for each of our loci. Phenotypes shown reached a p-value of genome-wide significance level (0.05×10^{-6}).
