# Supplementary material for: Scalable probabilistic PCA for large-scale genetic variation data
Source: PLoS Genet. 2020 May 29;16(5):e1008773. doi: 10.1371/journal.pgen.1008773 (PMC7286535; doi:10.1371/journal.pgen.1008773)
Supplement: S9 Table — We queried the Global Biobank Engine for associations from our loci. The Global Biobank Engine contains GWAS results for many more phenotypes than those available in the UK Biobank. Phenotypes shown are significant at genome-wide significance level (0.05 × 10^−6^). (PDF) [file pgen.1008773.s022.pdf]

SNP	Genes in Window	Phenotype Code	P	Phenotype
rs12913832	HERC2	INI1737	6.61E-24	Childhood_sunburn_occasions
rs492602	FUT2	INI3064	1.28E-09	Peak_expiratory_flow_(PEF)
		INI50	2.00E-11	Standing_height
		HC269	1.55E-14	high_cholesterol
		HC273	4.35E-08	essential_hypertension
		HC357	3.96E-10	duodenal_ulcer
		INI1289	4.42E-09	Cooked_vegetable_intake
		INI20015	1.51E-08	Sitting_height
		INI24019	3.36E-09	Particulate_matter_air_pollution_(pm10);_2007
		HC188	4.85E-17	cholelithiasis/gall_stones
		HC215	8.22E-13	hypertension
		HC225	6.19E-14	cholecystitis
rs5743614	TLR10,TLR1,TLR6,FAM114A1	HC382	4.97E-11	asthma
		HC49	1.18E-14	hayfever/allergic_rhinitis
		INI24019	1.25E-64	Particulate_matter_air_pollution_(pm10);_2007
rs62389423	IRF4,EXOC2	INI30120	1.62E-11	Lymphocyte_count
		INI30150	1.17E-14	Eosinophill_count
		INI30210	4.74E-08	Eosinophill_percentage
		INI50	1.26E-08	Standing_height
		INI134	3.57E-09	Number_of_self-reported_cancers
		INI1737	8.62E-164	Childhood_sunburn_occasions
		INI1873	6.06E-18	Number_of_full_brothers
		INI24004	1.66E-12	Nitrogen_oxides_air_pollution;_2010
		INI24006	7.33E-17	Particulate_matter_air_pollution_(pm2.5);_2010
		INI24017	6.46E-13	Nitrogen_dioxide_air_pollution;_2006
		cancer1003	2.41E-87	skin_cancer
		cancer1060	2.05E-99	non-melanoma_skin_cancer
		FH1001	3.98E-18	Lung_cancer
rs7570971	RAB3GAP1,R3HDM1,LCT	INI3062	1.11E-08	Forced_vital_capacity_(FVC)
		INI23100	1.37E-08	Whole_body_fat_mass
		INI24019	5.36E-12	Particulate_matter_air_pollution_(pm10);_2007
rs9267817	HLA	INI30100	1.36E-10	Mean_platelet_(thrombocyte)_volume
		INI30150	3.16E-16	Eosinophill_count
		INI46	1.31E-09	Hand_grip_strength_(left)
		INI50	1.51E-24	Standing_height
		HC303	5.00E-49	malabsorption/coeliac_disease
		INI20015	1.63E-14	Sitting_height
		INI21002	7.44E-12	Weight
		INI23098	2.31E-11	Weight
		INI24019	1.33E-10	Particulate_matter_air_pollution_(pm10);_2007
		FH1065	7.46E-11	High_blood_pressure
		HC215	3.20E-10	hypertension

Table S9: **Selection hits are associated with phenotypes from the Global Biobank Engine.** We queried the Global Biobank Engine for associations from our loci. The Global Biobank Engine contains GWAS results for many more phenotypes than those available in the UK Biobank. Phenotypes shown are significant at genome-wide significance level (0.05×10^{-6}).
